# Fiber scaffold bioartificial liver therapy relieves acute liver failure and extrahepatic organ injury in pigs

**DOI:** 10.7150/thno.58515

**Published:** 2021-06-11

**Authors:** Jun Weng, Xu Han, Fanhong Zeng, Yue Zhang, Lei Feng, Lei Cai, Kangyan Liang, Shusong Liu, Shao Li, Gongbo Fu, Min Zeng, Yi Gao

**Affiliations:** 1 Department of Hepatobiliary Surgery II, Guangdong Provincial Research Center for Artificial Organ and Tissue Engineering, Guangzhou Clinical Research and Transformation Center for Artificial Liver, Institute of Regenerative Medicine, Zhujiang Hospital of Southern Medical University, Guangzhou 510515, China.; 2 State Key Laboratory of Organ Failure Research, Southern Medical University, Guangzhou 510515, China.; 3 Department of Medical Oncology, Jinling Hospital, First School of Clinical Medicine, Southern Medical University, Nanjing 210000, China.; 4 Guangdong Qianhui Biotechnology Co., Ltd., Guangzhou 510285, China.

**Keywords:** liver failure, bioartificial liver, regeneration, multiorgan failure, hepatic encephalopathy

## Abstract

**Rationale:** Acute liver failure (ALF) causes severe liver injury and a systemic inflammatory response, leading to multiorgan failure with a high short-term mortality. Bioartificial liver (BAL) therapy is a promising approach that is hampered by the lack of appropriate bioreactors and carriers to retain hepatic cell function and poor understanding of BAL treatment mechanisms in ALF and extrahepatic organ injury. Recently, we used a fiber scaffold bioreactor (FSB) for the high-density, three-dimensional (3D) culture of primary porcine hepatocytes (PPHs) combined with an absorption component to construct a BAL and verified its function in a D-galactosamine (D-gal)-induced ALF porcine model to evaluate its protective effects on the liver and extrahepatic organs.

**Methods:** Male pigs were randomized into standard/supportive therapy (ST), ST+no-cell BAL (ST+Sham BAL) and ST+BAL groups and received treatment 48 h after receiving a D-gal injection. Changes in blood chemistry and clinical symptoms were monitored for 120 h. Tissues and plasma were collected for analysis by pathological examination, immunoblotting, quantitative PCR and immunoassays.

**Results:** PPHs cultured in the FSB obtained sufficient aeration and nutrition for high-density, 3D culture and maintained superior viability and functionality (biosynthesis and detoxification) compared with those cultured in flasks. All the animals developed ALF, acute kidney injury (AKI) and hepatic encephalopathy (HE) 48 h after D-gal infusion and received corresponding therapies. Animals in the BAL group showed markedly improved survival (4/5; 80%) compared with those in the ST+Sham BAL (0/5; p < 0.001) and ST (0/5; p < 0.001) groups. The levels of blood ammonia and biochemical and inflammatory indices were alleviated after BAL treatment. Increased liver regeneration and attenuations in the occurrence and severity of ALF, AKI and HE were observed in the ST+BAL group compared with the ST (p = 0.0009; p = 0.038) and ST+Sham BAL (p = 0.011; p = 0.031) groups. Gut leakage, the plasma endotoxin level, bacterial translocation, and peripheral and neuroinflammation were alleviated in the ST+BAL group compared with those in the other groups.

**Conclusions:** BAL treatment enhanced liver regeneration and alleviated the systemic inflammatory response and extrahepatic organ injury to prolong survival in the ALF model and has potential as a therapeutic approach for ALF patients.

## Introduction

Acute liver failure (ALF) is a life-threatening disease complicated by multiorgan failure, with a survival rate of less than 70% 1-3. Because of the limitations of liver donations, alternative therapies are needed to bridge patients to self-recovery or liver transplantation. Artificial liver devices, including mechanical artificial liver (MAL) (plasma exchange and toxin absorption) and bioartificial liver (BAL) (ammonia detoxification and biological secretion) devices, have been developed and tested in preclinical and clinical studies 4-9. The MAL device is mainly based on dialysis and corrects blood abnormalities, such as high levels of damage-associated molecular patterns (DAMPs) and inflammatory cytokines, improving the patient's mental status and circulatory stability, but not the patient's survival, likely because of the lack of metabolic detoxification, indicating that the complexity of liver functions is unlikely to be replaced by MAL alone 1, 3, 5, 6. In addition to the detoxification of the aforementioned substances, BAL devices containing hepatocytes possess the capacity for metabolic detoxification and synthetic activity and can successfully lower the blood ammonia level to prevent brain edema and improve survival in both D-gal- and hepatectomy-induced ALF 7, 9-11.

ALF-induced extrahepatic organ/system dysfunction is the most characteristic and lethal complication 12-17. The exact pathophysiology of extrahepatic organ injury in ALF remains elusive. The release of DAMPs from hepatocytes and systemic inflammatory responses (SIRs) are the most important factors for ALF and the evolution of extrahepatic organ injury, such as kidney and gut injury 18-22. Acute kidney injury (AKI), as the most common ALF-induced extrahepatic organ injury, is more likely to be classified as an inflammatory injury featuring tubular damage 16, 20, 23-25. Additionally, hyperammonemia and frequently coexistent AKI reduce renal ammonia excretion, further increasing the blood ammonia level and directly leading to neuronal dysfunction and cerebral edema, called hepatic encephalopathy (HE), which is associated with a mortality rate of more than 50% 26, 27. Hyperammonemia and SIRs cause neuroinflammation-mediated cognitive impairment and aggravate HE 28-30. Gastrointestinal injury (GI) is characterized by intestinal barrier damage resulting in bacterial translocation (BT) and the release of endotoxin and ammonia into the circulation 17. Immune cells such as Kupffer cells (KCs), activated by BT and lipopolysaccharide (LPS) through Toll-like receptors, release many inflammatory cytokines that worsen the permeability and structure of the intestines, further activating KCs and resulting in a vicious cycle that ultimately induces liver and extrahepatic organ failure 13, 31, 32. BAL therapy focused on the early stage of ALF between 12 and 24 h has exhibited superior ability to reduce inflammation and enhance liver regeneration to improve survival 4, 11, 33, 34. Because of the rapid progression of ALF, in most admitted patients, ALF is complicated with different degrees of extrahepatic organ failure and beyond the early stage 35. Whether BAL therapy could relieve liver and extrahepatic organ failure to improve the prognosis and the possible mechanism require further study.

Therefore, we adapted a BAL using a double plasma molecular adsorption system (DPMAS) as the MAL component and a fiber scaffold bioreactor (FSB) as the BAL component to achieve both absorption and detoxification. The safety and efficacy of the BAL were evaluated in a randomized experiment using a D-gal-induced ALF porcine model. We showed that BAL treatment prolonged animal survival while protecting the liver and extrahepatic organs and alleviating the inflammatory response. BAL therapy resulted in strong liver regeneration: hepatocytes were reprogrammed to the fetal stage and showed a low rate of apoptosis; an intact intestinal barrier inhibited BT and endotoxin release; little AKI was detected because of slight renal tubular injury and TLR4 activation. Additionally, decreased levels of ammonia and neuroinflammation were observed in BAL-downregulated brain injury.

## Materials and Methods

### Group setting

Healthy male (35-47 kg; 1-2 years old) Tibetan miniature pigs (Animal Center of Southern Medical University, Dongguan, China) were randomly divided into a supportive/standard therapy (ST; n = 5) group, an ST with BAL without cells group (ST+Sham BAL; n = 5), and an ST with BAL group (ST+BAL, n = 5). All the animal experimental protocols were approved by Southern Medical University Animal Care and Use Committee (Approval No. 2016063A) and followed the guidelines of the Laboratory Animal Welfare Act and amendments thereof.

### Porcine ALF model establishment and standard therapy

Following arrival, the pigs received anesthesia (30 mg/kg of sodium pentobarbital and 0.15 mL/kg of Xylazine hydrochloride injection) to place two double-lumen hemodialysis access catheters (11-F; 20 cm; ABLE, Guangzhou, China), one in the right internal jugular vein and one in the femoral vein; these catheters were then used to collect blood samples and maintain anesthesia. Once injected with D-gal (T = 0 h), the animals were allowed to recover from anesthesia. The catheter was locked with heparin saline (2500 U/mL) to maintain patency. Clinical observation was performed every 6 h during the experiment. Blood samples were collected every 12 h for chemical testing starting at t = 0 h (albumin [ALB], aspartate aminotransferase [AST], alanine aminotransferase [ALT], blood urea nitrogen [BUN], total bilirubin [TBIL], creatinine [Cr], glucose, ammonia) and coagulation function testing (DRI-7000i, FUJI and URIT). All the samples were stored at -80 °C for further testing. An AST level exceeding 100 times the baseline level was referred to as “ALF”. ALF complications, AKI and HE diagnoses, and grading were evaluated according to previously described guidelines 14, 36-38. Different reported doses of D-gal (0.45, 0.5, 0.55, 0.6 and 0.65 g/kg) were used to determine the optimal-dose ALF model for BAL saving experiments, and the 0.45 g/kg ALF model was considered the most appropriate (Figure S1A) 7, 9, 10, 33, 34. All the animals showed symptoms of illness and fatigue and rapidly increased blood levels of AST, ALT, BUN, TBIL, Cr, and ammonia, as well as pathological abnormalities (Figure S1B-J). Pigs administered a dose higher than 0.45 g/kg rarely survived more than 48 h, which did not provide a sufficient treatment window. Pigs administered 0.45 g/kg showed significant ALF, HE and AKI within approximately 48 h and finally died within 72 h, providing a sufficient window to treat ALF and extrahepatic organ failure. Thus, 0.45 g/kg and 48 h were chosen as the dose and BAL treatment timepoint, respectively. Propofol was injected via a central venous catheter (0.15-0.2 mg/kg/min) to maintain slight anesthesia, and the blood pressure, heart rate, and respiratory rate were monitored during the BAL and sham BAL treatments. Animals in all the groups received 500 mL of normal saline before D-gal injection and treatment (t = 48 h). When the animals stopped eating and drinking because of ALF, they received 5% dextrose normal saline (DNS5) at 50 mL/h to satisfy their basic water and energy needs. Twenty milliliters of 50% dextrose were injected when the blood glucose level fell below 5 mmol/L. The experiment timeline is illustrated in Figure 2J.

### Study endpoints

The animals were followed for at least 120 h after D-gal injection. Animals with ALF that survived 120 h reached survival as the study endpoint. Animals that showed nonrecoverable cardiorespiratory arrest or stage IV HE (coma, nonresponse to an ear tug or pain stimulus and pupil dispersal) reached death as the endpoint. Animals that reached survival and started to show independent feeding without symptoms of HE (signs of recovery) were judged to have recovered and were euthanized by a propofol overdose for future experiments.

### Tissue examination

Animal tissues were collected after the endpoint, fixed and embedded in paraffin for hematoxylin and eosin (H&E) and immunohistochemical staining. Antibodies against Ki-67 (27309-1-AP; Proteintech), neutrophil gelatinase-associated lipocalin (NGAL) (GTX60965; GeneTex), TLR-4 (GTX75742; GeneTex), Epcam (Ab71916; Abcam), SOX9 (Ab185966; Abcam), AFP (A8452; Sigma), CK18 (Ab668; Abcam), ALB (Ab79960; Abcam), YAP (Ab81183; Abcam), IBA-1 (GT10312; GeneTex), CD206 (Ab64693; Abcam), TNF-α (Ab6671; Abcam), GFAP (ab7260; Abcam), F4/80 (ab6640; Abcam), Occludin (ab31721; Abcam), and ZO-1 (ab214228; Abcam), as well as the TUNEL staining kit (T2190; Solarbio) and Masson's trichrome kit (G1346; Solarbio) were used according to the manufacturers' instructions. Pathology section examinations were performed at least three times for each animal. H&E-stained liver sections were observed and scored by pathologists in a blinded fashion using a quantitative system (inflammation [absent, 0; present, 1]; zone 3 necrosis [absent, 0; present 1]; panlobular necrosis [absent, 0; focal, 1; diffuse, 2]; percentage necrosis [absent, 0; 1-25%, 1; 26-76%, 2; >75%, 3]; and fatty changes [absent, 0; present, 1]), as reported 7. Chilu's score and immunohistochemistry positive counts were calculated by two pathologists blinded to the sample groups, and the means of at least five random horizons of three different splices were employed for analysis. Positive results for nuclear staining by Ki-67 and TUNEL were counted and defined as the regenerative index and apoptotic index, respectively. Positive area (Masson; GFAP) and number (microglial perimeter; nuclear) calculations were conducted using ImageJ as reported previously 28, 30. Brain water content assays were performed after the animals were sacrificed, and samples were collected as reported previously 7. Cell and tissue samples were collected and stored in 2.5% glutaraldehyde for scanning electron and transmission electron microscope examinations as reported previously 7.

### Detection of plasma chemical, endotoxin and inflammatory factors

All the blood samples were collected and centrifuged at 4000 rpm for 10 min for plasma collection. Plasma chemicals were evaluated using biochemical instruments (DRI-7000i, FUJI and URIT). The change speed was calculated as follows: [Parameter (treatment end, 56 h)- Ammonia (treatment end, 48 h)]/treatment time (8 h). Cytokines were assessed using the Luminex 200 system and the Porcine Cytokine 13-plex Panel Magnetic Bead Kit (Luminex, Austin, USA). The plasma endotoxin concentrations were measured using the Endotoxin Detection Kit (B50-600L; LONZA) according to the manufacturer's instructions.

### RNA and protein assays

Total RNA was prepared from liver, kidney and intestine samples from all the groups using TRIzol (Invitrogen) and was reverse transcribed into cDNA using the iScript cDNA Synthesis Kit (Bio-Rad, Hercules, CA). RT-PCR was performed using the SsoFast Eva Green Supermix Kit (Bio-Rad) according to the instructions on the ABI 7300 RT-PCR platform (Primer sequence; Table S1). All the qPCR data were repeated three times, and the results were normalized to β-actin expression. RNA-seq was performed and assayed by Novogene (Beijing, China). The differentially expressed genes (DEGs) were calculated and labeled using the “Limma” package. Subsequently, DEGs were analyzed by gene ontology (GO) and Kyoto Encyclopedia of Genes and Genomes (KEGG) pathway analyses. GO analysis comprises three parts: molecular function (MF), biological process (BP), and cell component (CC). The results were statistically significant at the less than 0.05 level using a p-value. The tissues from each animal were homogenized with RIPA buffer. Proteins were separated by SDS-PAGE and transferred to nitrocellulose membranes, which were incubated with primary antibodies against Occludin (ab31721; Abcam), β-catenin (ab16051; Abcam), E-cadherin (ab15148; Abcam) or GAPDH (10494-1-AP; Proteintech). Horseradish peroxidase (HRP)-conjugated goat anti-rabbit or anti-mouse IgG (SA00001-1, SA00001-2; Proteintech) was used as the secondary antibody. The relative protein expression was determined using HRP-conjugated secondary antibodies and electrochemiluminescent (ECL) substrates. The intensities of the immunoreactive bands were quantified by densitometry using Image Lab software, and the target protein expression levels were normalized to that of GAPDH and calculated the relative fold change to the ST group. AKI-associated plasma proteins were assessed using an AKI antibody array (ab169806; Abcam) according to the manufacturer's instructions.

### Bioreactor design and function information

**Figure 1:**
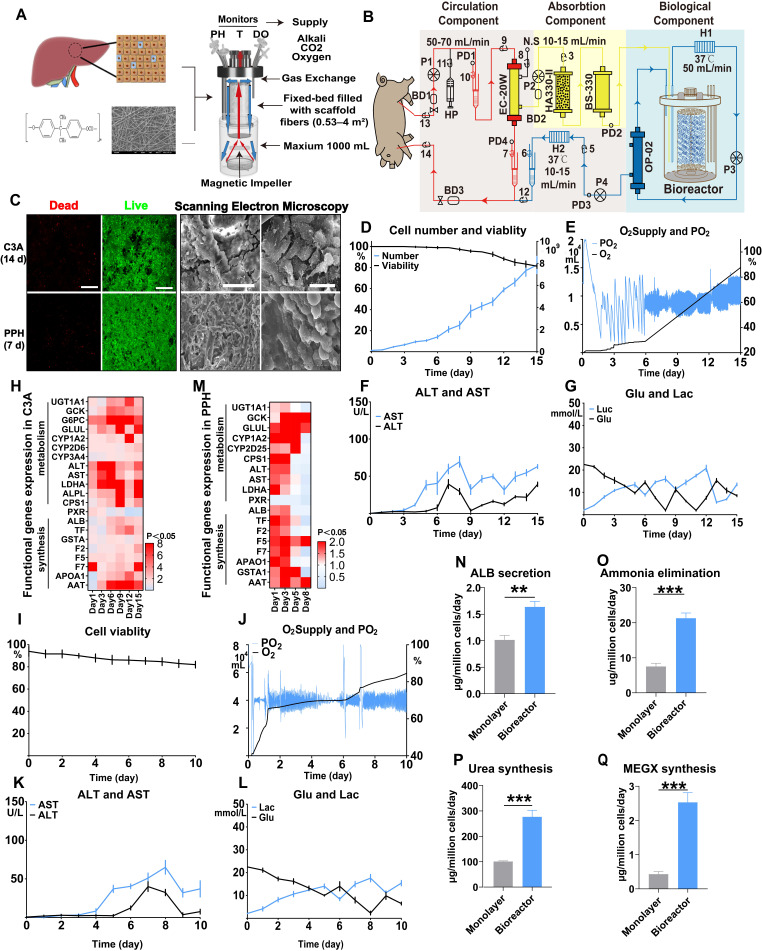
** Hepatocytes maintain cell viability and function under high-density, 3D culture in the bioreactor. A.** The bioreactor contained fiber scaffolds made of PET. The pH, temperature and dissolved oxygen were monitored, and alkali, carbon dioxide (CO_2_) and oxygen (O_2_) were automatically added to the bioreactor to maintain a stable culture environment. The culture medium was vertically transported by a magnetic impeller (red arrow) and flowed as a thin film down the outer wall like a “waterfall” from the top, enabling O_2_/CO_2_ stripping without bubbles harming the cells. The medium volume ranged from 600 to 1000 mL depending on the cell number. The speed of the magnetic impeller was calculated using a medium speed and volume. **B.** Structural diagram of the FSB BAL therapy system. The red line identifies the route of blood circulation at a speed of 50 mL/min and pressure of 100 mmHg powered by a blood pump (BP). Heparin was injected at a baseline of 500 U/h to maintain the APTT between 175 s and 250 s (HP). The yellow and blue lines represent the plasma separated by a hollow fiber membrane plasma separator (EC-20W; 80% rejection rate at 100 kDa), which occurred at a speed of 15-25 mL/min and a pressure of 50-70 mmHg powered by pump P1. The nonbiological component represented by the yellow area contained active carbon and a bilirubin adsorption column (HA330-II; BS330; Jianfan, China). After the absorption step, the plasma entered the biological component and was detoxified by hepatocytes, followed by transport, powered by pump P2, to the second hollow fiber membrane plasma separator (OP-02; 330 µm) to block potentially exfoliated cells and fragments. Finally, purified plasma was returned to the animal at a speed of 50 mL/min and a pressure of 50-70 mmHg powered by pump P3. A bubble detector was used to monitor microbubbles (BD1, BD2 and BD3), which were removed by a venous chamber. The system pressure was monitored using a pressure detector (PD1, PD2, PD3 and PD4). Extranormal saline was injected at 10-15 mL/min as the baseline. To maintain the temperature of circulation, a heater (H1 and H2) provided additional heating. **C.** Live/dead detection of the viability of C3A cells (14 d) and PPHs (5 d), which remained more than 80% viable (Bar = 200 µm). Scanning electron microscopy (SEM) of C3A cells (14 d) and PPHs (5 d) revealed that the C3A cells and PPHs attached to the fibers and filled the space between the fibers (Bar = 100 µm, Bar = 20 µm, n = 3 per group). **D.** Cell number and viability of C3A cell culture from 0 to 15 d (n = 3 per time point). **E.** Oxygen supply and dissolved oxygen (PO_2_) of C3A cell culture from 0 to 15 d (n = 3 per time point). **F.** ALT and AST levels of C3A cell culture from 0 to 15 d (n = 3 per time point). **G.** Glucose and lactate levels of C3A cell culture from 0 to 15 d (n = 3 per time point). **H.** C3A cell functional gene expression after 15 d of culture (normalized to the corresponding levels of C3A cells cultured in flasks; p < 0.05; n = 3 per time point). **I.** Viability of PPH cells from 0 to 10 d (n = 3 per time point). **J.** Oxygen supply and dissolved oxygen (PO_2_) in PPH cells cultured from 0 to 10 d (n = 3 per time point). **K.** ALT and AST levels in PPH cells cultured from 0 to 10 d (n = 3 per time point). **L.** Glucose and lactate levels in PPH cells cultured from 0 to 10 d (n = 3 per time point). **M.** PPH functional gene expression after 10 d of culture (normalized to the corresponding levels of day-1 PPHs cultured in flasks, p < 0.05; n = 3 per time point). **N.** ALB secretion of day-1 PPHs cultured in the bioreactor (p = 0.0012; n = 3 per time point). **O.** Ammonia elimination of day 1 PPHs cultured in the bioreactor (p < 0.001; n = 3 per time point). **P.** Urea synthesis of day-1 PPHs cultured in the bioreactor (p < 0.001; n = 3 per time point). **Q.** CYP450 metabolic activity of day-1 PPHs cultured in the bioreactor (assayed by monoethylglycinexylidide (MEGX) synthesis; p < 0.001; n = 3 per group).

The bioreactor was designed to provide adequate oxygen exchange, a sufficient nutrient supply and a three-dimensional (3D) culture mode for hepatocytes (112 mm, 135 mm). The middle of the bioreactor was a fixed bed (100 mm, 200 mL) with fiber scaffolds made of medical-grade polyester microfibers (11.3 cm^2^ per piece), providing a growth surface maximum of 4 m^2^. Evenly distributed media circulation was achieved using a magnetic drive impeller (red arrow), ensuring low shear stress and high cell viability. The culture medium flows through fiber scaffolds from the bottom to the top. At the top, the medium falls as a thin film down the outer wall, where it takes up O_2_ and releases CO_2_ to maintain high PO_2_ and low PCO_2_ in the bioreactor (blue arrow) (Figure 1A, Figure S2A, and Table S2). This unique waterfall oxygenation, together with gentle agitation and biomass immobilization, enables the compact bioreactor system to achieve and maintain high cell densities-equaling the productivity of much larger stirred-tank units. The pH, temperature and dissolved oxygen were monitored, and alkali, carbon dioxide (CO_2_) and oxygen (O_2_) were automatically added to the bioreactor to maintain a stable culture environment. The medium volume ranged from 600 to 1000 mL depending on the cell numbers and culture mode. The magnetic impeller speed was calculated using a medium circulation speed and volume to maintain a linear velocity of less than 2 cm/s to ensure a low shear stress microenvironment (Table S3-4).

### Hepatocyte harvest and bioreactor culture

C3A cells, a subtype of HepG2 cells with higher ALB synthesis ability widely used in seed cell BAL studies, were adopted to evaluate the bioreactor effect on proliferative cells 39. C3A cells were cultured in MEM supplemented with 10% FBS and 1× penicillin and streptomycin. Cell viability was determined using a Live/Dead Viability Kit (L3224; Thermo Fisher) according to the manufacturer's instructions. Cell numbers were determined by lysis of the cell membrane to calculate the number of nuclei in at least three fiber scaffolds. Healthy donor Tibetan miniature pigs (15-20 kg; age <1 year) were obtained from Southern Medical University. Twenty-four hours before treatment, the bioreactor system (iCellis nano; Pall) was precultured with serum-free hepatocyte culture medium HepatoZYME-SFM (17705021; Gibco; 1000 mL). Twelve hours before BAL treatment, hepatocytes were harvested according to a previously reported 2-step perfusion method 7. The viability of the isolated hepatocytes was determined by Trypan blue staining. Primary hepatocytes were seeded in the bioreactor at a perfusion speed of 2 cm/s (1094 rpm) for 2 h. After the cells adhered to the fiber scaffold, the perfusion speed was maintained at 1 cm/s (673 rpm). PPHs were plated on a Matrigel-coated culture dish (AN-572; Corning) at 1.5 × 105 cells/cm^2^ using HepatoZYME-SFM as a control. During the whole process, the temperature, pH and percentage of dissolved oxygen (PO_2_) were maintained at 37.3 °C, 7.3±0.1 and 65%±10%, respectively (Figure 1A). The cell number and vitality were determined before treatment.

### Hepatocytes and BAL function examination

To examine PPH ALB secretion, the supernatants of the bioreactor and plate culture were collected after 24 h and analyzed using an ALB enzyme-linked immunosorbent assay (ELISA) kit (ab108794; Abcam). Ammonia elimination and urea synthesis were assayed by incubating PPHs with 3 mM NH_4_Cl in HepatoZYME-SFM. NH_4_^+^ and urea in the supernatant collected 24 h after induction were measured using enzymatic colorimetric assays (Megazyme International). For the CYP450 metabolic activity assay, monoethylglycinexylidide (MEGX) synthesis was performed, as reported previously 40. For BAL function evaluation, we employed simulated ALF serum assays (5 g/L ALB, 660 mmol/L ammonia, 5.0 mmol/L urea, 450 µmol/L Cr, 140 µmol/L TBIL and 140 µmol/L TBA in RPMI 1640 medium), and the supernatants during BAL culture (0, 2, 4, 8, 10 h) were collected and measured using a biochemical instrument (DRI-7000i; FUJI). All the assays were performed in at least three repetitions.

### Extracorporeal therapy (ECT)

ECT using the sham BAL or BAL device was initiated at 48 h. Propofol (0.10 mg/kg/min) was used to sedate animals during the treatment. The heart rate, blood pressure and cutaneous oximetry monitoring were performed during ECT. The sham BAL and BAL devices were connected to the pigs with induced ALF via a central venous catheter (Figure 1B and Figure S3). Five hundred milliliters of saline were injected before treatment to prevent possible hypotension. DNS5 (50 mL/h) was continuously infused during treatment, with an increase to the maximum rate of 300 mL/h to maintain the blood pressure above 60/30 mmHg. Blood chemistry and coagulation function were tested every 2 h during treatment. Heparin was injected to maintain the activated partial thromboplastin time (APTT) between 175 s and 250 s.

### Statistical analysis

All the data are presented as means ± SD. Unpaired two-tailed Student's-test and analysis of variance (ANOVA) were employed to test the statistical significance of protein and gene expression. The Mantel-Cox log-rank test was performed to assess the survival time. P < 0.05 was considered statistically significant. Statistical analysis was performed using GraphPad Prism 8.

## Results

### The bioreactor culture maintains hepatocyte viability and function at a high density

The fiber scaffolds (11.2 cm^2^ surface area per piece) comprised medical-grade polyester microfibers (PET); each bioreactor provided a maximum growth surface area of 4 m^2^ once supplemented with 500-1000 mL of medium. The fiber scaffolds were fixed in place from the middle, and the culture medium was pumped up through the scaffolds from the bottom to the top. The medium flowed down the outer wall in a thin film similar to a waterfall, enabling oxygenation/CO_2_ stripping without bubbles harming the cells (Figure 1A). After 15 d, 1.5×10^8^ C3A cells cultured on the 4 m^2^ surface increased to 93×10^8^, with a cell viability exceeding 80% (Figure 1C), a sufficient oxygen and nutrient supply (Figure 1D), and mild cell damage (Figure 1E-G). The functional gene expression of these hepatocytes was 2- to 10-fold greater than that of cells cultured in flasks (Figure 1H). Next, we cultured 60×10^8^ primary porcine hepatocytes (PPHs) in a bioreactor for 10 d. The bioreactor provided a sufficient supply of necessary components and a stable environment, allowing a high cell survival rate (more than 80%) and low cell injury (ALT, AST) to be maintained (Figure 1C, I-L). PPHs in the bioreactor activated pathways associated with ECM secretion and hepatocyte polarity reestablishment, likely identifying a 3D culture mode (Figure S2C-E). During an 8 d culture period, the PPHs maintained relatively high expression levels of most hepatocyte functional genes (such as GCK, CPS1, TF, LDHA, GSTA1, GLUL, F5, and F7) (Figure 1M). PPHs in the bioreactor (day 1) showed a significantly higher level of oxygen consumption, indicating that they were in an active metabolic mode (Figure 1J). ALB secretion, ammonia elimination, urea synthesis and CYP-drug metabolism were greater in the bioreactor culture than in the flask culture, also confirming that day-1 PPHs might be the most advantageous for BAL treatment (Figure 1N-Q). These observations indicate that the FSB enabled high-density, 3D culture of hepatocytes with great metabolic and synthetic functionality.

### Pre-evaluation of the BAL therapeutic effect *in vitro*

**Figure 2:**
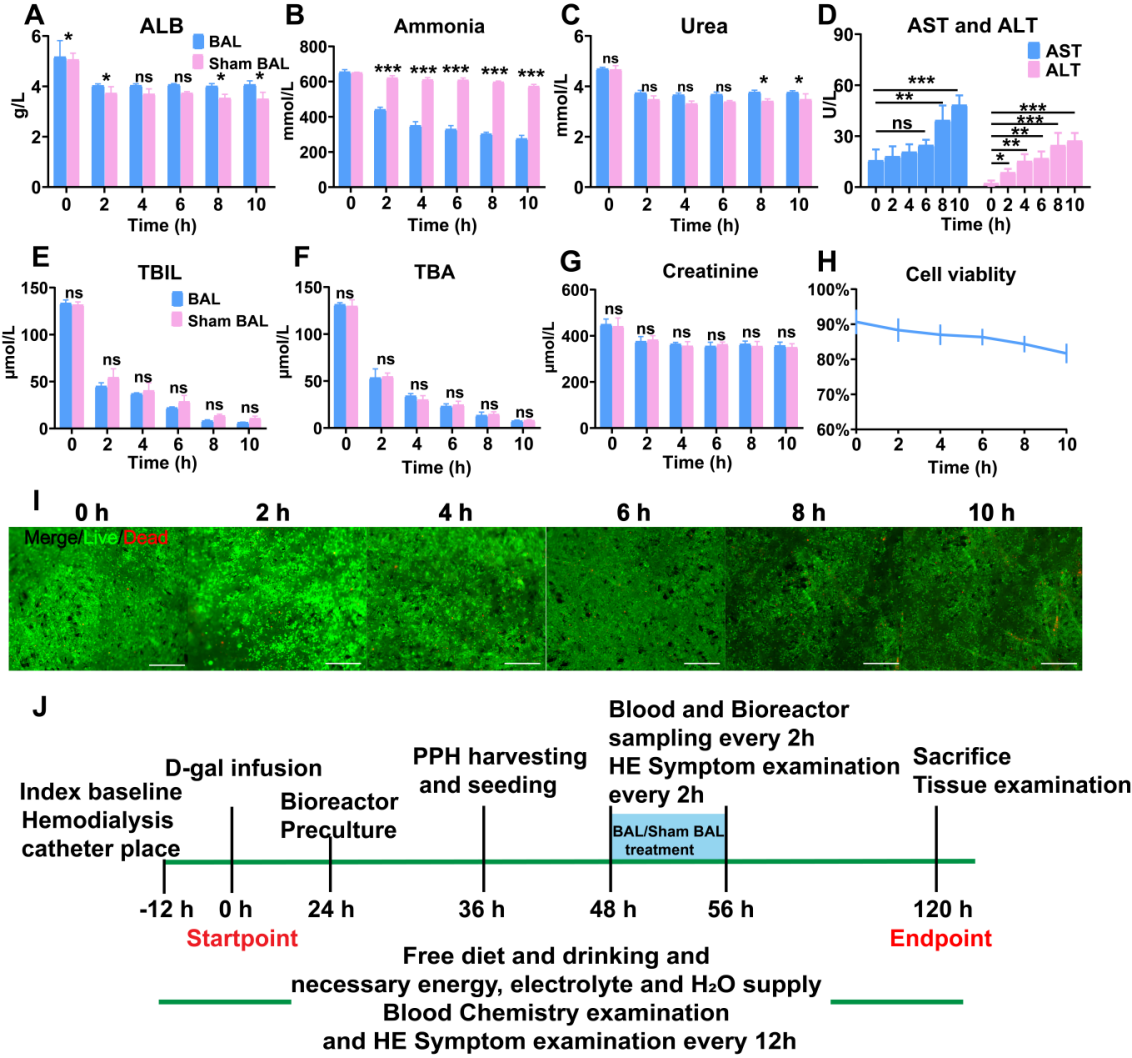
** BAL device and therapeutic efficacy *in vitro*. A.** ALB, **(B)** ammonia, **(C)** urea, **(D)** ALT, **(E)** and AST, **(F)** TBIL, **(G)** TBA and **(H)** Cr levels in simulated ALF serum (p < 0.05, p < 0.001; n = 3). **H.** PPH vitality in the BAL device over 10 h of circulation (n = 3 per time point). **I.** Live/dead assay of PPHs in the BAL device over 10 h of circulation (n = 3 per time point). **J.** *In vivo* experimental procedure. Pigs had undergone placement of two double-lumen hemodialysis access catheters, blood testing and D-gal infusion at 0 h; the bioreactor was started at 24 h for preculture. PPHs were isolated and seeded at 36 h. ALF pigs had undergone BAL or sham BAL therapy at 48 h for 8 h, and the chemical indices in the blood and bioreactor were determined every 2 h. After treatment, the pigs were monitored until 120 h and then sacrificed, at which time blood and tissue were collected for further analysis.

The BAL device contained circulation, absorption and biological components (Figure 1B). To determine the effect of BAL therapy on ALF, we used BAL-containing PPHs and sham BAL therapy to purify and detoxify simulated ALF serum containing the main toxic substances in ALF (ammonia, TBIL, total bile acid (TBA)). After 10 h of circulation, the BAL group showed significantly increased ALB and urea levels and a decreased ammonia level, indicating profound ammonia detoxication via ureagenesis (p < 0.05, p < 0.01, p < 0.001; Figure 2A-C). The Sham BAL and BAL groups both showed decreases in the TBIL and TBA levels to quite low levels; however, no Cr reduction was found (Figure 2E-G). These toxins slightly reduced the cell viability as the ALT and AST levels increased, but the overall viability remained higher than 80% (Figure 2D, H, I). These results indicated that BAL therapy could maintain enhanced cell viability and detoxify toxic substances without protein loss, showing therapeutic potential for ALF. We next tested the BAL device in an ALF porcine model (Figure 2J and Figure S3).

### Performance of BAL therapy in preventing ALF progression

**Figure 3:**
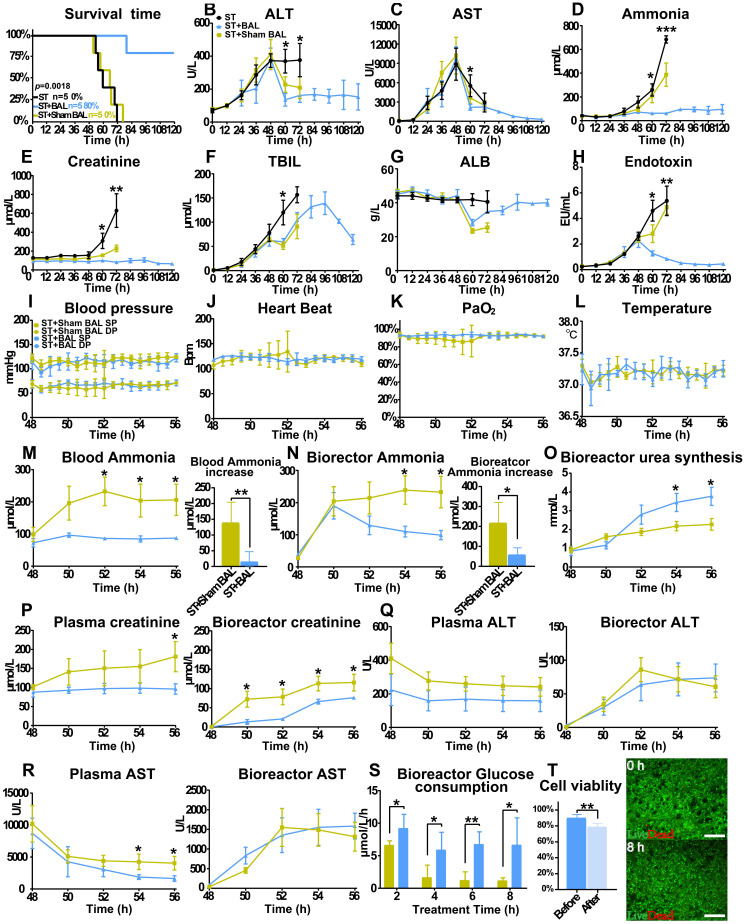
** BAL therapy prevents ALF progression by detoxification. A.** Survival time in the ST+BAL, ST+Sham BAL and ST groups (n = 5 per group; log-rank test).** B-H.** Blood biochemistry parameters in the three groups during the experiment (ALT, AST, ammonia, Cr, TBIL, ALB, endotoxin; n = 5 per group; p < 0.05, p < 0.01, p < 0.001; n = 5 per group). **I-L.** Vital signs in the three groups during treatment (blood pressure, heart beat, blood oxygen and temperature; n = 5 per group). **M-O.** Ammonia and Urea metabolism during treatment (p < 0.05, p < 0.01; n = 5 per group). **P-R.** Blood biochemistry parameters in the three groups during treatment in the bioreactor and blood (Cr, ALT and AST levels; p < 0.05; n = 5 per group). **S.** PPH glucose consumption every 2 h during treatment (p < 0.05; p < 0.01; n = 5 per group). **T.** PPH live/dead staining at the beginning and end of treatment (p < 0.01; n = 5 per group).

All the animals started to lose their appetite after 24 h, followed by progression to ALF, HE (stage I to II) and AKI, with comparable laboratory values at 48 h. Animals in the ST+BAL group showed improved survival (4/5; 112±17.9 h; 80%) compared with those in the ST+Sham BAL (0%; 62.4±8.2 h; p < 0.001) and ST groups (0%; 64.0±9.5 h; p < 0.001) (Figure 3A). The ALT and AST levels started to decrease after 48 h, with lower levels in the ST+BAL group (Figure 3B, C). The blood ammonia and Cr levels remained at relatively stable, low levels with no stage IV HE or AKI in the ST+BAL group compared with those in the ST and ST+Sham BAL groups (Figure 3D, E and Table 1). The TBIL level showed a late increase and returned to normal after BAL treatment compared with the other treatments (Figure 3F). The ALB and endotoxin levels recovered to normal after BAL treatment, indicating liver regeneration and no future hepatocyte damage (Figure 3G, H). Except for one pig in the ST+Sham BAL group that died because of nonrecoverable cardiorespiratory arrest (HRA) during treatment, all the pigs tolerated the treatment procedure with stable vital signs (Figure 3I-L).

Next, we examined toxicity in the blood and bioreactor during treatment to classify the metabolic detoxification ability of the therapy. The ammonia level in the blood slightly increased after BAL treatment compared with sham BAL treatment, indicating that BAL therapy partly replaced original liver function (p < 0.05; Figure 3M and Figure S4A). The bioreactor ammonia level decreased after a slight increase and remained low in the BAL group compared with that in the sham BAL group (p < 0.05; Figure 3N and Figure S4B). Additionally, urea accumulation significantly increased in the BAL group compared with that in the no-cell BAL group, indicating that PPHs in the bioreactor metabolized ammonia into urea (p < 0.05; Figure 3O and Figure S4C-D). The Cr levels in the bioreactor and blood remained relatively low in the BAL group compared with that in the sham BAL group, indicating renal protective effects during treatment (p < 0.05; Figure 3P). Although the ALT and AST levels decreased in both groups, the BAL group showed a lower AST level with a higher ALB level, suggesting stronger liver protection with BAL therapy (p < 0.05; Figure 3Q-R and Figure S4E-F). The plasma TBIL level was similar in the two groups, likely because of its longer physiological metabolism time than the treatment; the TBIL level was undetected in the bioreactor because of the profound absorption effect (Figure S4G). The glucose consumption in the BAL group maintained a more stable speed than that in the sham BAL group, indicating that PPHs maintained a stable detoxification ability (Figure 3S). Although PPH viability slightly decreased after treatment (89.8%±4.4% vs 78.6%±4.3%; p = 0.004), the average viability was still greater than 70%, suggesting that BAL therapy could be repeated or continued for longer durations (Figure 3T).

### BAL therapy alleviates liver injury and enhances liver regeneration

**Figure 4:**
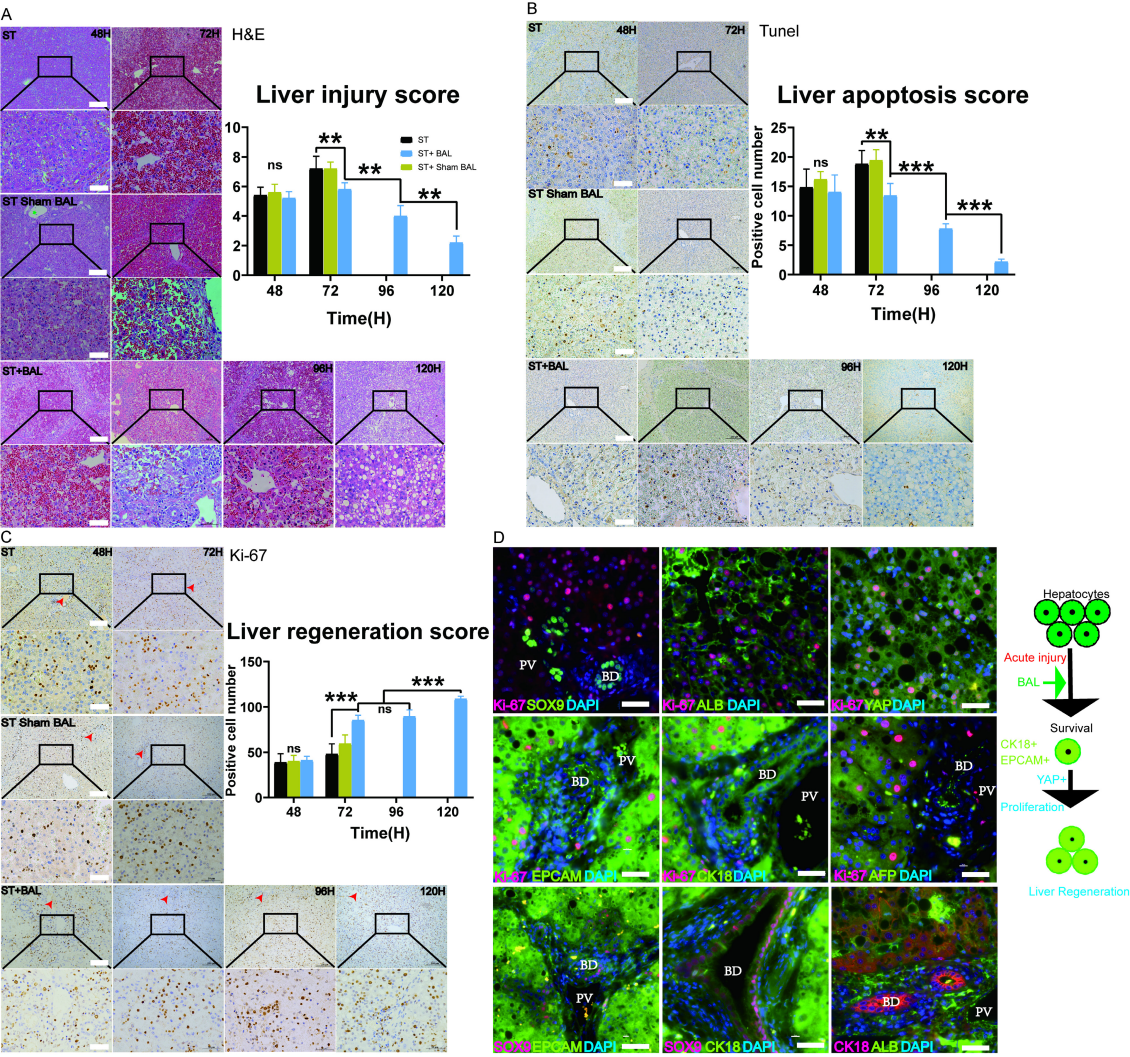
** BAL therapy alleviates liver injury and enhances regeneration. A-B.** H&E staining of liver tissue from the ST, ST+Sham BAL and ST+BAL groups (higher magnification images are shown in the insets below). Liver injury was scored (p < 0.01; Bar = 200 µm, Magnification Bar = 50 µm; n = 5 per group). **C-D.** TUNEL staining of liver tissue from all the groups (higher magnification images are shown in the insets below). The number of positive cells per 40× field was calculated for at least 4 separate tissue sections per pig (p < 0.01, p < 0.001; Bar = 200 µm; Magnification Bar = 50 µm; n = 5 per group). **E-F.** Ki-67 staining of liver tissue from all the groups (higher magnification images are shown in the insets below; red arrows represent bile ducts). The number of positive cells per 40× field was calculated for at least 4 separate tissue sections per pig (p < 0.001; Bar = 200 µm; Magnification Bar = 50 µm; n = 5 per group). **G.** Ki-67, SOX9, CK18, Epcam, YAP, AFP and ALB co-immunostaining of the liver from the ST+BAL group at 120 h (BD represents Bile Duct, PV represents Portal Vein area; Bar = 40 µm). **H.** Schematic of hepatocyte proliferation and regeneration.

Liver histology and immunohistochemistry were performed to observe ALF progression. H&E staining at 48 h showed hemorrhage, hepatocyte vacuolar damage and apoptosis in all the groups. Animals in the ST and ST+Sham BAL groups showed progression to extensive necrosis with bleeding throughout the lobules and prominent inflammation at 72 h; however, those in the BAL group showed significantly alleviated liver damage, with a lower liver injury score than animals in the other groups (p < 0.01; Figure 4A). Increased liver parenchymal cells were observed at 96 h and 120 h, indicating liver regeneration after treatment (Figure 4A). TUNEL staining was employed to evaluate liver apoptosis. Similar positive cell staining was observed in the three groups at 48 h, while the ST+BAL group showed a lower level at 72 h that continued to decrease until the endpoint, indicating that BAL prevented future hepatocyte apoptosis (p < 0.01, p < 0.001; Figure 4B). The bile ductular reaction is associated with liver regeneration and inflammation 41, 42. Masson's staining in the three groups showed the same extent of increase in the bile zone at 48 h; by contrast, the staining intensity was slightly decreased in the ST+BAL group and remained at that level until the endpoint (p < 0.05; Figure S5A). To examine hepatocyte proliferation and regeneration, we analyzed the expression of the proliferation marker Ki-67. Ki-67-positive hepatocytes appeared in all the groups at 48 h, concentrated in the portal vein area rather than the bile zone (Figure 4E-F). The number of Ki-67-positive hepatocytes was much higher in the ST+BAL group than in the ST and ST+Sham BAL groups, and this level remained stable and high until the end of the experiment, indicating that BAL therapy strongly enhanced proliferation (p < 0.001; Figure 4F). To further understand the hepatocyte proliferation mechanism, we examined classical regeneration pathways in the ST+BAL group. Immunostaining and serial sectioning showed that areas of Ki-67 and SOX9 expression were not colocalized, possibly indicating that the regenerative cell source was mainly residual hepatocytes rather than ductal progenitors (Figure 4G and Figure S5C-D). Immunostaining also showed that hepatocytes strongly coexpressed Ki-67 with CK18, Epcam, and YAP, indicating their dedifferentiation into an immature state, possibly providing the proliferative capacity for liver regeneration (Figure 4G). Unlike the strong expression of ALB, hepatocytes showed weak AFP expression on staining, indicating that the dedifferentiation was not completely reversed to an immature state. Notable ductular reactions were detected by AFP, SOX9, Epcam and CK18 (stem cell markers) in the bile zone, which also identified the existence of ductal progenitors and their exchange to a more fatal state in the bile zone (Figure 4H). Together, these results indicate that BAL therapy alleviates liver injury and progression and strongly enhances hepatocyte regeneration by dedifferentiation.

### BAL therapy alleviated peripheral inflammation

**Figure 5:**
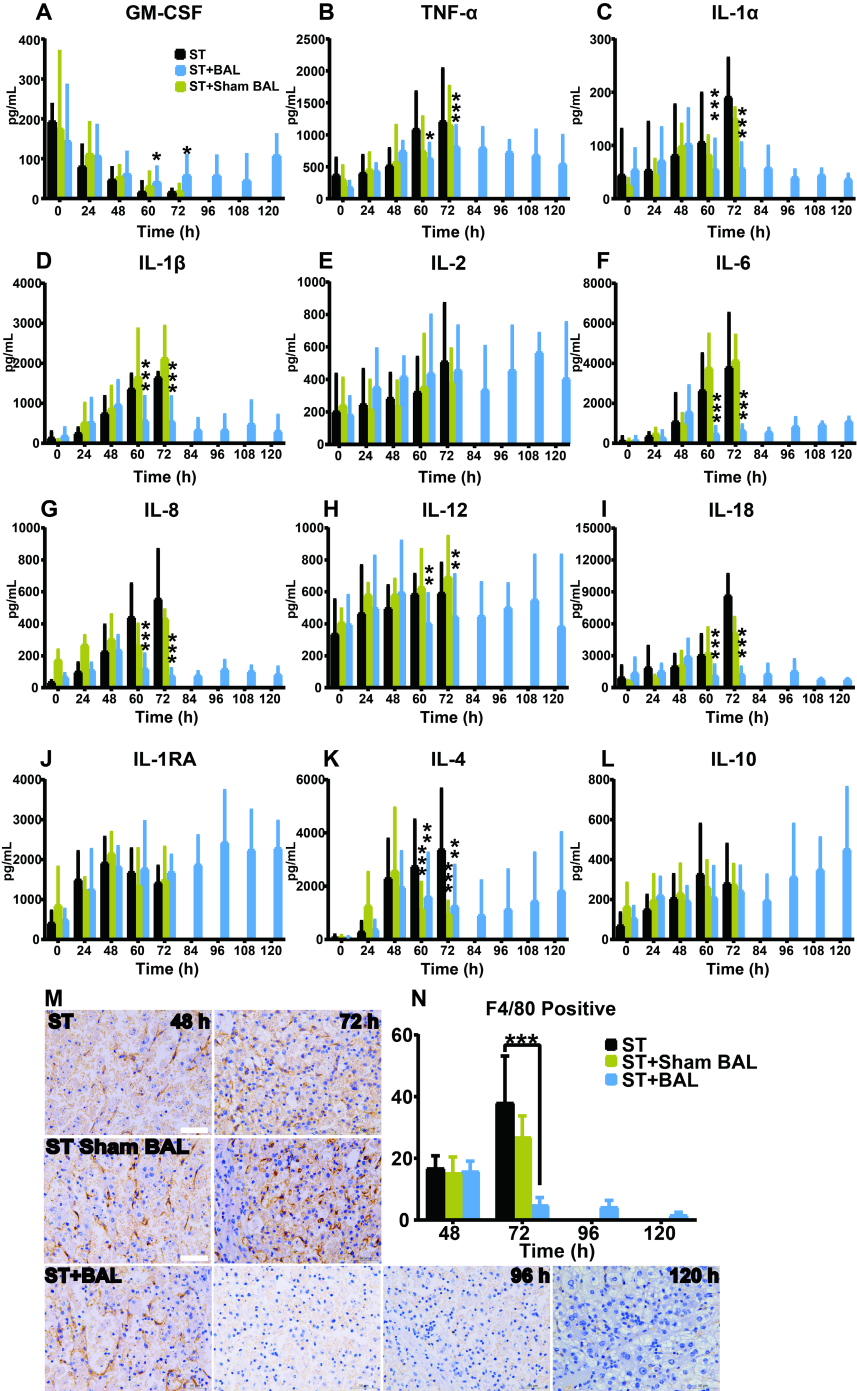
** BAL therapy alleviates peripheral inflammation. A-L.** Plasma inflammatory factor assay results, including GM-CSF, TNF-α, IL-1α, IL-1β, IL-2, IL-6, IL-8, IL-12, IL-18, IL-1RA, IL-4 and IL-10 (p < 0.05, p < 0.01, p < 0.001; n = 5 per group). **M.** F4/80 staining of KCs in all the groups (n = 5 per group). **N.** F4/80 calculation per 40× field for at least 4 separate tissue sections per pig (p < 0.001; n = 5 per group).

ALF occurs with a strong inflammatory response featuring the activation of immune cells, marked increases in proinflammatory cytokines, and progression to extrahepatic organ injury and dysfunction 43, 44. The levels of inflammatory cytokines (TNF-α, IL-1α, IL-1β, IL-2, IL-6, IL-8, IL-12, and IL-18) and anti-inflammatory cytokines (IL-1RA, IL-4, and IL-10) were similar from 0 h to 48 h, and a gradual increase in the inflammatory response was observed along with ALF progression (p < 0.05, p < 0.01, p < 0.001; Figure 5A-L). Regarding the GM-CSF level, all the groups showed a decreasing trend, while the BAL group showed a reverse trend after treatment, likely indicating its positive relationship with survival (Figure 5A) 11. After BAL treatment, the TNF-α, IL-1α, IL-1β, IL-6, IL-8, IL-12, and IL-18 levels declined to varying degrees compared with those after ST and ST+Sham BAL treatment; by contrast, only the level of the anti-inflammatory factor IL-4 decreased after BAL and Sham BAL treatment, indicating that BAL therapy might alleviate the inflammatory response by downregulating proinflammatory factors rather than increasing counter regulatory factors (Figure 5A-L). KCs, as the main source of the immune response and origin of related inflammatory factors, were examined by F4/80 staining; a sharp decrease in KC activity was observed after BAL treatment compared with the other treatments, and this decrease persisted until the end of treatment (p < 0.001; Figure 5M, N). Furthermore, Ki67 and TUNEL staining identified that the KCs were in a relatively more proliferative state in ST and ST+Sham BAL compared with that in the BAL group (Figure S6A-B). These results suggest that BAL alleviates both the peripheral inflammatory response and subsequent inflammation activation/damage/activation in a viscous cycle.

### BAL therapy protects intestinal permeability and alleviates gut leakage, BT and endotoxin release

**Figure 6:**
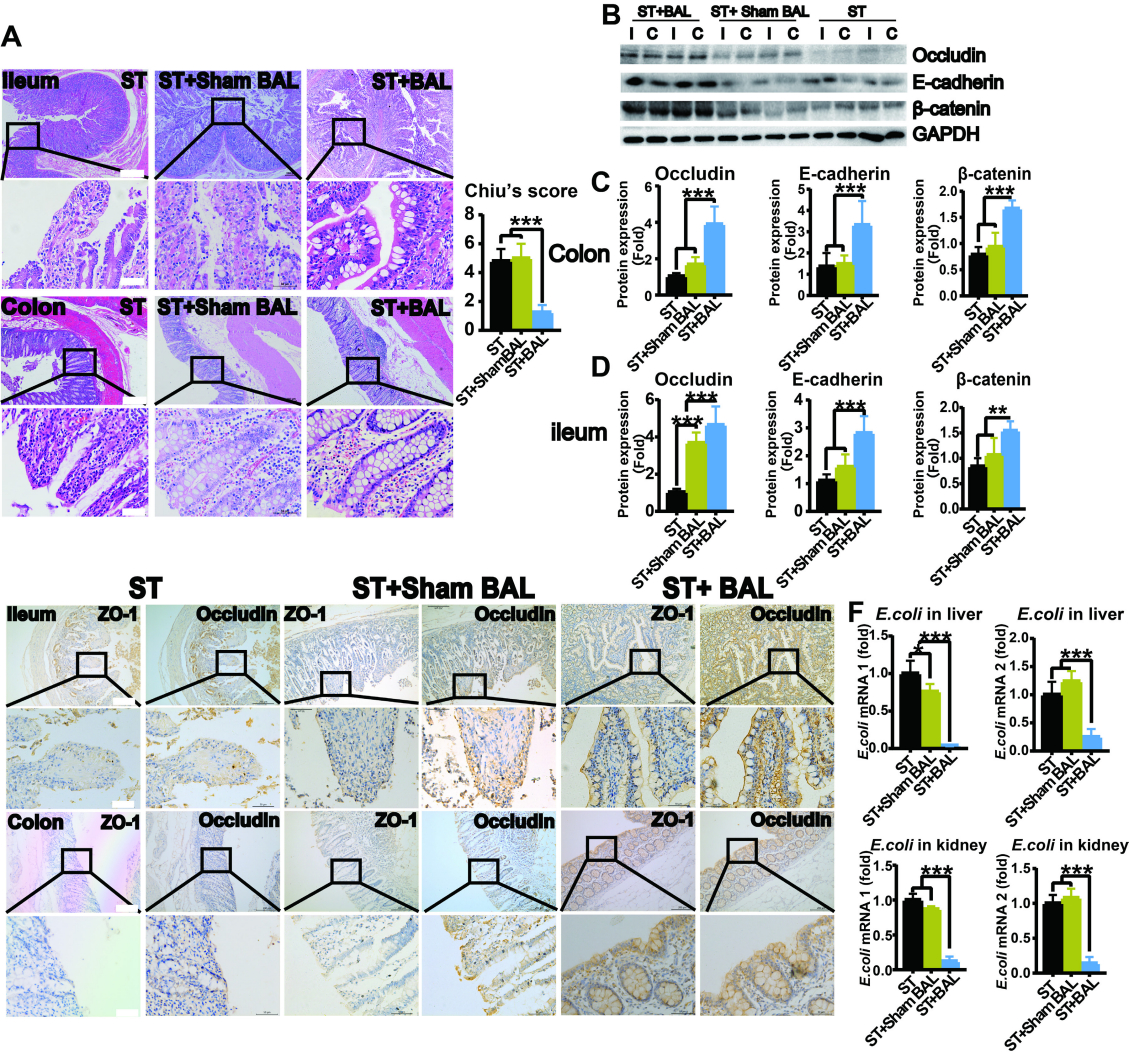
** BAL therapy protects intestinal permeability. A.** H&E staining of ileum and colon tissue from all the groups at the end of the experiment and Chilu's score for ileum injury (higher magnification images are shown in the insets below; (p < 0.001; Bar = 200 µm; Magnification Bar = 50 µm; n = 5 per group). **B.** Western blotting of occludin, E-cadherin, β-catenin, and GAPDH expression in ileum and colon tissue from all the groups at the end of the experiment. **C.** Relative occludin, E-cadherin, and β-catenin protein expression in the ileum (p < 0.01, p < 0.001; n = 5 per group). **D.** Relative occludin, E-cadherin, and β-catenin protein expression in the colon (p < 0.01, p < 0.001; n = 5 per group). **E.** Immunohistochemistry of serial sections of the ileum and colon for ZO-1 and Occludin (higher magnification images are shown in the insets below; Bar = 200 µm; Magnification Bar = 50 µm; n = 5 per group). **F.** *E. coli* expression in the liver and kidney (p < 0.05, p < 0.001; n = 5 per group).

Intestinal histological analysis revealed a marked loss of the epithelium covering the surface of the intestines, a disorderly glandular arrangement, glandular dissolution and increased inflammatory cells with interstitial infiltration in the ileum and colon in the ST and ST+Sham BAL groups compared with those in the BAL group, with an increased injury score (p < 0.001; Figure 6A). Intestinal tight junction proteins (Occludin) and adherent junction proteins (E-cadherin and β-catenin), which are critical for maintaining the integrity and function of the intestinal barrier, showed significantly decreased expression in the ileum and colon in the ST and ST+Sham BAL groups compared with that in the BAL group (p < 0.001, p < 0.01; Figure 6B-D). To further examine the intestinal barrier, serial sections were employed to observe ZO-1 and Occludin staining, which was weaker than that in the ST+BAL group (Figure 6E). *Escherichia coli* (*E. coli*) and endotoxin are typical toxins in gut leakage; we checked the *E. coli* mRNA levels in the liver and kidneys at the end of the experiment, and they were elevated in the ST and ST+Sham BAL groups compared with those in the BAL group (p < 0.0001, Figure 6F). The plasma endotoxin level was elevated in all the groups at 48 h and markedly decreased after BAL treatment (p < 0.001, p < 0.05; Figure 3H). Consistently, these findings indicate that BAL therapy alleviates GI, which may increase the levels of BT and plasma endotoxin and may act as a factor promoting severe inflammatory reactions in ALF.

### BAL therapy alleviates inflammation and kidney injury

**Figure 7:**
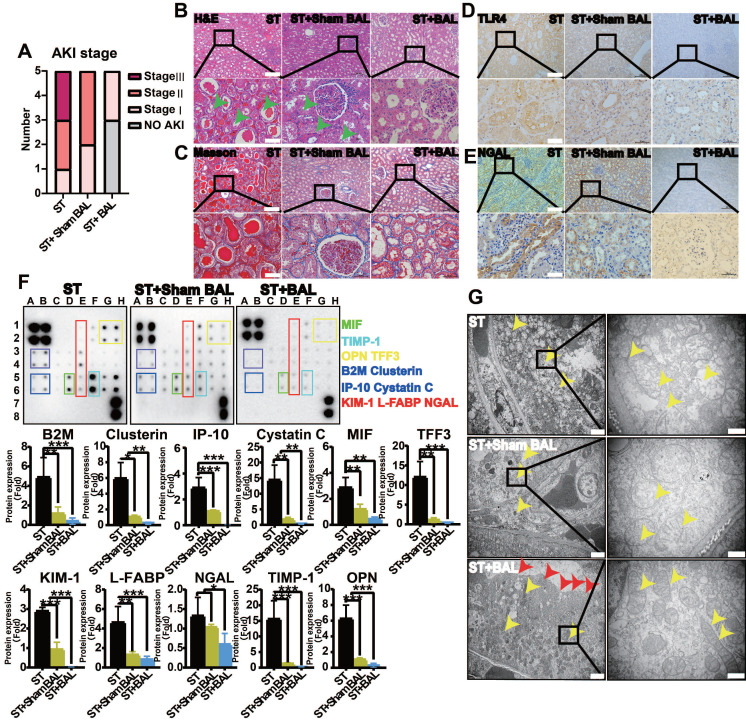
** BAL therapy alleviates inflammation and kidney tubular injury. A.** AKI stage in all the groups at the endpoint (n = 5 per group). **B.** H&E staining of kidney tissue at the endpoint (green arrows indicate the edematous and dilated tubules; higher magnification images are shown in the insets below; Bar = 200 µm; Magnification Bar = 50 µm). **C.** Masson's staining of kidney tissue at the endpoint (higher magnification images are shown in the insets below; Bar = 200 µm; Magnification Bar = 50 µm). **D.** TLR4 staining of kidney tissue at the endpoint (higher magnification images are shown in the insets below; Bar = 200 µm; Magnification Bar = 50 µm). **E.** NGAL staining of kidney tissue at the endpoint (higher magnification images are shown in the insets below; Bar = 200 µm; Magnification Bar = 50 µm). **F.** AKI-associated plasma protein assay at 72 h (NGAL, OPN, MIF, IP-10, clusterin, KIM-1, L-FABP, cystatin C, B2M, TTF3, and TIMP-1; p < 0.05, p < 0.01, p < 0.001; n = 5 per group). **G.** TEM observation of the kidney at the endpoint (yellow arrow indicates swelling mitochondria; red arrow indicates the brush border; higher magnification images are shown in the insets below; Bar = 2 µm; Magnification Bar = 500 nm).

AKI is the most common complication in ALF, with a mortality rate of nearly 60%, and is likely an indicator of inflammatory kidney injury, including acute tubular injury (called non-hepatorenal syndrome [nonHRS]-AKI), rather than HRS 15, 16, 20, 45. We examined the severity of kidney injury and expression of related inflammatory factors. At 72 h, the Cr level was significantly lower in the ST+BAL group than in the ST (p = 0.038) and ST+Sham BAL (p = 0.031) groups (Figure 3E). Although the ST+Sham BAL group had a lower Cr level than the ST group, no significant difference was found (p = 0.15; Figure 2E). Animals in the ST group had higher levels of AKI (2 stage Ⅲ, 2 stage Ⅱ and 1 stage Ⅰ) than those in the ST+Sham BAL (3 stage Ⅱ and 2 stage Ⅰ) and ST+BAL (2 stage Ⅰ and 3 no AKI) groups (Figure 7A). Pathological sections showed tubular injury in the form of edematous and dilated tubules containing protein casts in the ST and ST+Sham BAL groups compared with that in the ST+BAL group, while the glomerulus maintained a normal morphology in all the animals (Figure 7B). No significant fibrosis was observed in any group on Masson's staining (Figure 7C), indicating that kidney injury was mainly tubular. TLR4, the main inflammatory kidney injury marker in sepsis and SIRs 21, 46, was extensively expressed in tubules in the ST and ST+Sham BAL groups compared with that in the ST+BAL group (Figure 7D). Staining for the kidney injury marker NGAL revealed extensive expression in tubules in the ST and ST+Sham BAL groups compared with that in the ST+BAL group, suggesting that kidney injury was focused on the tubules (Figure 7E). The expression levels of AKI-associated plasma proteins, including the proinflammatory mediators NGAL, osteopontin (OPN), macrophage migration inhibitory factor (MIF), interferon gamma-induced protein-10 (IP-10) and clusterin, the structural proteins kidney injury molecule-1 (KIM-1) and liver-type fatty acid-binding protein (L-FABP), the tubular-reabsorbed proteins cystatin C, beta-2-microglobulin (B2M), and trefoil factor 3 (TTF3), and the cell cycle regulatory protein tissue inhibitor of metalloproteinases-1 (TIMP-1), were markedly elevated in the ST and ST+Sham BAL groups compared with those in the BAL group (p < 0.05, p < 0.01, p < 0.001; Figure 7F). Transmission electron microscopy (TEM) of tubular epithelial cells from the ST group showed that the tubules were severely injured and dilated, the brush border was diminished, and the mitochondria had ruptured or disappeared. In the ST+Sham BAL group, the mitochondria in the tubular cells maintained their basic shape, with swelling and broken cristae. Tubular cells in the ST+BAL group showed the least damage; the brush border was maintained, with slight swelling of the mitochondria and an intact crista structure (Figure 7G). Overall, plasma inflammatory factors, endotoxemia and BT, as the main inflammatory factors, were significantly alleviated in the BAL group, possibly reducing the kidney inflammatory response and induction of tubular injury.

### BAL therapy reduces neuroinflammation and alleviates HE

**Figure 8:**
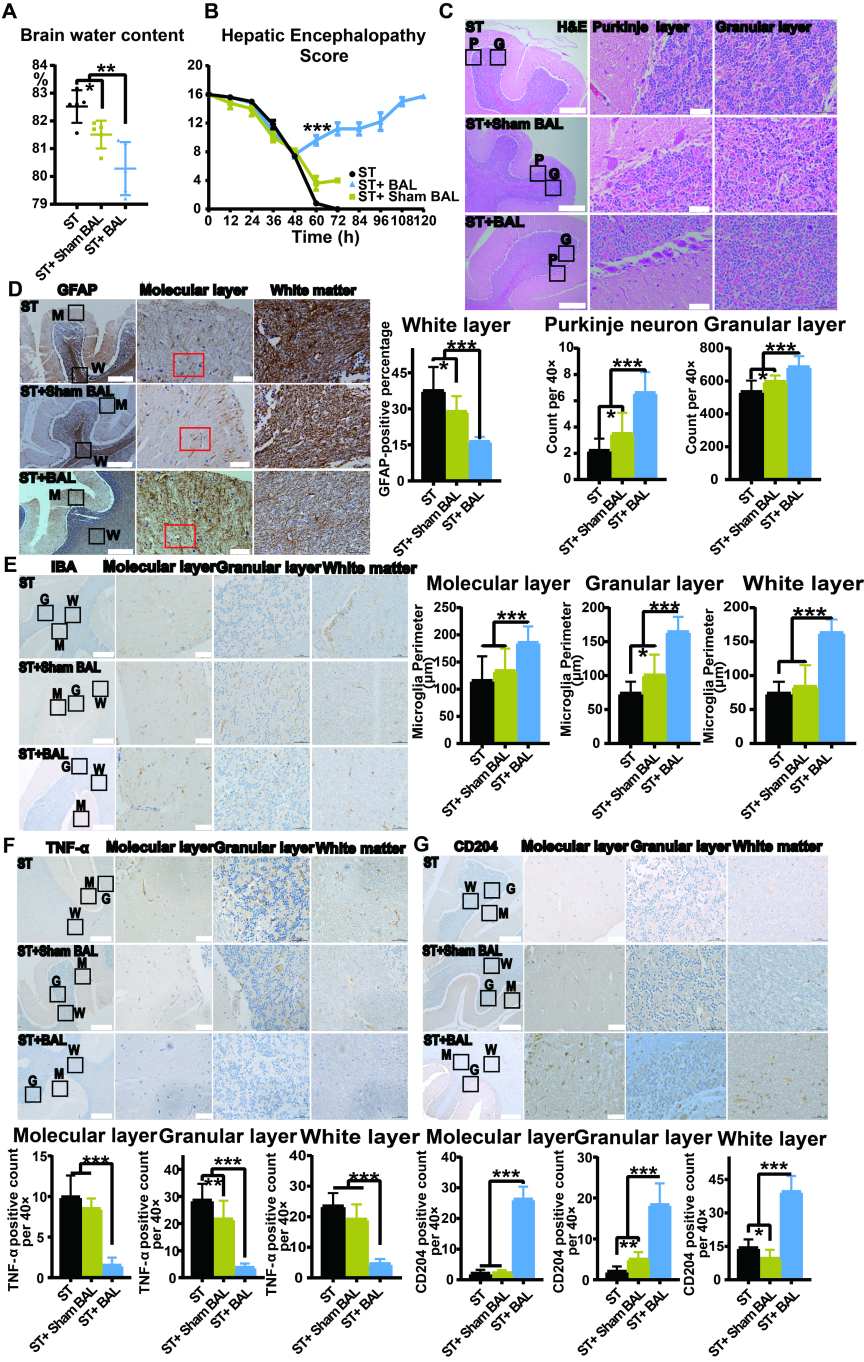
** BAL therapy alleviates neuroinflammation and HE. A.** Brain water content in all the animals at the endpoint (p < 0.05, p < 0.01; n = 5 per group). **B.** HE score in all the animals during the experiment (p < 0.001; n = 5 per group). **C.** H&E staining of the cerebellum (P represents the Purkinje layer; G represents the granular layer; higher magnification images are shown in the right insets; Bar = 200 µm; Magnification Bar = 50 µm; p < 0.05, p < 0.001; n = 5 per group). **D.** GFAP staining of the cerebellum; the GFAP-positive area was calculated and compared (M represents the molecular layer; G represents the granular layer; W represents the white layer; higher magnification images are shown in the right insets; Bar = 200 µm; Magnification Bar = 50 µm; p < 0.05, p < 0.01; n = 5 per group). **E.** IBA staining of the cerebellum; the microglial perimeter was calculated and compared (M represents the molecular layer; G represents the granular layer; W represents the white layer; higher magnification images are shown in the right insets; Bar = 200 µm; Magnification Bar = 50 µm; p < 0.05, p < 0.01; n = 5 per group). **F.** TNF-α staining of the cerebellum; the number of positive cells per 40× field was calculated and compared (M represents the molecular layer; G represents the granular layer; W represents the white layer; higher magnification images are shown in the right insets; Bar = 200 µm; Magnification Bar = 50 µm; p < 0.01, p < 0.01; n = 5 per group). **G.** CD204 staining of the cerebellum; the number of positive cells per 40× field was calculated and compared (M represents the molecular layer; G represents the granular layer; W represents the white layer; higher magnification images are shown in the right insets; Bar = 200 µm; Magnification Bar = 50 µm; p < 0.05, p < 0.01, p < 0.001; n = 5 per group).

HE features cerebral edema and neuronal dysfunction, often induced by hyperammonemia 29, 47. In addition to directly causing cerebral edema, hyperammonemia also induces neuroinflammation, mediating cognitive impairment 28. The brain water content was used to evaluate cerebral edema and was lowest in the ST+BAL group compared with that in the other groups (p = 0.0012), while the brain water content was lower in the ST+Sham BAL group than that in the ST group (p = 0.0192; Figure 8A). We used the HE score to examine neuronal function during the experiment. In the first 48 h, the score decreased in all the groups. After 8 h of treatment, the score was markedly increased in the ST+BAL group compared with that in the other groups at 60 h (p < 0.001; Figure 8B). The score in the ST+BAL group recovered to normal and remained in that state for the rest of the experiment, indicating that both neuronal function and cerebral edema were maintained better in the ST+BAL group than in the other groups (Figure 8B). The cerebellum, as one of the first and most seriously damaged areas in HE, was used to examine HE progression 47, 48. H&E staining revealed neuronal loss in both the Purkinje and granular layers in the ST and ST+Sham BAL groups compared with that in the ST+BAL group, possibly explaining the HE score decline (Figure 8C). Neuroinflammation is mainly induced by the activation of astrocytes and microglia 49. To evaluate astrocytic activation, we examined GFAP expression in the molecular layer, and the ST+BAL group showed a significantly lower GFAP-positive area than the other groups (p < 0.05, p < 0.001; Figure 8D). The perimeter of microglia specifically decreases with activation and differentiation 50. The microglial perimeter in the molecular, granular and white layers in the ST and ST+Sham BAL groups was significantly reduced compared with those in the ST+BAL group, indicating greater activation in the ST and ST+Sham BAL groups (p < 0.001; Figure 8E). Once activated, microglia differentiate into a proinflammatory (M1) state to exacerbate tissue damage or an anti-inflammatory (M2) state to facilitate tissue repair 51. To determine the cell state, we employed TNF-α for M1 and CD206 for M2 staining, and the number of M1 (TNF-α) cells was significantly lower in the ST+BAL group (p < 0.001; Figure 8F). By contrast, the number of M2 (CD206) cells was markedly upregulated in the ST+BAL group (p < 0.001) compared with that in the other group, with a higher number in the ST+Sham BAL group than in the ST group (p = 0.0468; Figure 8G). These findings suggest that BAL therapy has a significant protective effect on the brain by alleviating cerebral edema neuroinflammation.

## Discussion

ALF occurs because of abrupt hepatocyte injury and features liver dysfunction symptoms and extrahepatic organ failure 35. BAL devices offer a potential approach for bridging patients to transplantation or self-recovery 52. The therapeutic efficacy of BAL mainly relies on sufficient functional cells and supportive bioreactor design, which are the greatest challenges in BAL development 1, 3, 5, 6. Different cell sources have been employed for BAL devices, such as primary human hepatocytes (PHHs) 53, human HCC cell lines 54, 55, induced human hepatocytes 9, 34, and porcine primary hepatocytes (PPHs) 7, 11, 56-59. PHHs are the most ideal cell source for BAL devices; however, because of donor shortage and preservation means, PHHs cannot serve as a stable and clinically applicable hepatocyte source. HCC cell lines, such as C3A, have been employed in extracorporeal liver assist devices (ELADs) for clinical trials 55, 60. Although unlimited proliferation ability provides stable and accessible cell sources, a lack of normal hepatocyte functions, such as ammonia metabolism, limits the BAL treatment effect in hyperammonemia and HE 44, 61. Induced human hepatocytes derived from fibroblasts 9, mesenchymal stem cells 61, induced pluripotent stem cells 33 and liver progenitor-like cells 34 were applied in BAL devices and showed profound treatment effects in ALF models to improve survival. However, the complex acquisition process and time requirement could not meet the urgent and rapid demand of ALF patients. PPHs are the most convenient and fastest hepatocyte sources for BAL 58. Furthermore, their similar synthesis and detoxification functions to human hepatocytes granted PPHs more possibilities in BAL treatment 62, 63. However, PPH BAL HepatAssist using a hollow fiber bioreactor failed in clinical trials 64. In the most widely used hollow fiber bioreactors, cell culture and medium perfusion are on separate sides, ensuring a low-shear stress two-dimensional (2D) culture environment but significantly degrading mass transfer efficacy and limiting hepatocyte three-dimensional (3D) structure formation, possibly explaining BAL failure in improving patient survival 44, 57-60, 62. Facing this shortage, Glorioso successfully employed a spheroid reservoir bioartificial liver (SRBAL) to culture PPH spheres and alleviate ALF in a porcine model 4, 11 and clarified that an adequate oxygen exchange, a sufficient nutrient supply and three-dimensional (3D) culture are critical determinants of bioreactors to support BAL therapy 10, 39. However, central anoxia and relatively large sphere culture volumes limit its clinical translation 7, 11. Facing unavoidable plasma toxic effects to hepatocytes, a hybrid BAL may help maintain hepatocyte viability and assure a treatment effect 54, 58, 65.

Therefore, we employed the FSB, which provides a 4 m^2^ culture surface area with a 1000 mL culture volume; the FSB circulated medium from the bottom to the top without dead space, and a high O_2_/CO_2_ exchange efficiency was achieved by liquid flowing down without bubble-induced shear force. At an experimental clinical scale (10^9^-10^10^), hepatocytes were successfully cultured at a high density in the bioreactor with viability exceeding 80% at 10 d. Additionally, the hepatocytes formed 3D structures and maintained increased functionality in albumin synthesis and ammonia metabolism, which are limited in tumor-derived or immortalized cell lines. Unlike in sphere or organoid formation, continuous gentle circulation of the medium shortens the seeding and adhesion process to at least 2 h, providing the possibility of rapid and immediate BAL preparation in 4 h or less (including PPH acquisition and culture). The liquid flow mode also ensures direct mass transport and *in vivo*-like liver blood flow for metabolism and detoxification. Overall, the FSB supports functional, clinical-scale, 3D hepatocyte culture with an efficient O_2_ supply and mass transport.

ALF induces hyperammonemia, jaundice, coagulopathy, and hypoglycemia and progresses to multiorgan failure and death 7, 9, 11, 56. A simple MAL device can absorb TBIL and some inflammatory cytokines but fails to alleviate complications and improve survival 4, 7, 8, 39. Combining MAL and BAL devices successfully improves the survival rate of the ALF porcine model. HE, AKI and GI are the most common and severe forms of extrahepatic organ failure 1, 5, 7, 8, 18. HE, the life-threatening complication of ALF models, was alleviated by direct detoxification of ammonia during BAL treatment 4, 33, 34. Regarding AKI and GI, because of late occurrence and concealment, sufficient notice has not been obtained 17, 20.

HE syndrome was observed in all the animals at 48 h; however, the HE score stopped deteriorating and recovered to normal after BAL treatment. At the same time, ammonia, the key driver of cerebral edema in the BAL group, decreased to the baseline level, indicating the ability of the BAL device to remove ammonia. Consistent with the urea synthesis and glucose consumption in the bioreactor, significantly increased ammonia removal was dependent on the hepatic urea cycle. The HE score and ammonia level remained at a relatively lower level after BAL therapy compared with those reported previously 4, 33, 34, indicating its ability to enhance rather than simply replace residual hepatocyte regeneration and function. Neuroinflammation has been reported to mediate cognitive impairment and aggravate HE 28, 38, 66. Pathological examination illustrated downregulated astrocyte and microglial activation with an increase in M2-type (anti-inflammatory) microglia, suggesting an additional neuroinflammation alleviation effect of our BAL devices.

Strong and lasting hepatocyte regeneration is crucial for ALF recovery 28-30. BAL therapy significantly stimulates hepatocyte regeneration, as proven by the increasing number of Ki-67-positive hepatocytes. The proliferation of hepatocytes in ALF is increased mainly in the portal vein area rather than in the bile zone. Immunohistochemistry illustrated that BAL therapy promotes hepatocyte regeneration by activating the YAP pathway to promote dedifferentiation and reacquisition of the ability to proliferate. In contrast to regeneration in chronic liver injury, which depends on ductal reactions, this process results in rapid regeneration with the retention of basic hepatocyte features to guarantee basic liver function, possibly explaining the late decrease in TBIL 41, 67. Drug-induced hepatotoxicity mainly occurs within 24 h, and subsequent liver injury occurs mainly because of inflammation and shock. BAL therapy alleviates the progression of inflammation, possibly ameliorating liver injury and reducing apoptosis 68-70. Active regeneration may relieve inflammation and subsequent damage and change the balance toward liver recovery, which is likely the primary reason for improved survival.

DAMPs released from extensive hepatocyte death activate the immune system and cause a SIR, with increases in inflammatory factors, such as TNF-α and IL-6, and IL-8 24, 45, 71. These proinflammatory cytokines mediate gut ischemia and cell junction protein degradation to cause gastrointestinal barrier damage, leading to BT and endotoxin release, which further activate SIRs, resulting in a vicious cycle of aggravated liver and extrahepatic organ injury 18, 56. After BAL treatment, the levels of proinflammatory cytokines, including TNF-α, IL-1α, IL-1β, IL-6, IL-8, IL-12, and IL-18, were decreased and remained low compared with those after the other treatments. KCs, as the main immune cells, significantly decreased in activity after BAL treatment, suggesting that the lasting alleviation of the immune response was the result of reduced toxicity via liver regeneration. Immunohistochemistry suggested a relatively intact gastrointestinal barrier, with higher junction protein (ZO-1, Occludin, E-cadherin and β-catenin) expression. Consequently, the levels of BT and plasma endotoxin in the ST+BAL group were also reduced compared with those in the other groups. The plasma Cr level sharply increased after significant liver injury, and kidney pathology featured acute tubular injury with TLR4 and NGAL overexpression, indicating that the AKI in the D-gal-induced porcine model was secondary to the immune response and may be classified as nonHRS-AKI 21, 46, 72. BAL treatment prevented the increase in Cr and AKI severity compared with the other treatments. Additionally, the levels of the AKI plasma proteins in the BAL group remained lower than those in the other groups, indicating that BAL therapy provides protection from immune response-induced AKI in pigs. These findings confirm that BAL therapy alleviates the immune response and destroys the immune-gut-liver-extrahepatic organ cycle to reduce ALF and related complications.

This study has some limitations that should be acknowledged. ALF, inflammation, AKI, GI and HE are extremely complex disease processes that may be influenced by many factors. The drug-induced ALF porcine model may not fully reflect the physiology or mechanism of ALF. Acute-on-chronic liver failure (ACLF) and hepatectomy-induced ALF may exhibit different developmental mechanisms and characteristics. Different ALF models are necessary to understand the effect and mechanism of BAL therapy. Porcine models are the fastest, easiest and most economical source of functional hepatocytes; however, there is still the potential for xenozoonosis 11. However, with research on xenozoonosis-free animals, porcine hepatocytes could be a promising resource.

Overall, we developed an FSB that enabled the clinical-scale, 3D culture of functional hepatocytes, showing marked ability to support liver function by detoxifying ammonia, enhancing residual hepatocyte regeneration, alleviating inflammation, protecting extrahepatic organs and improving the recovery and survival of pigs in an ALF model.

## Supplementary Material

Supplementary figures and tables.

## Figures and Tables

**Table 1:** Treatment parameters and outcomes

Group		Treatment parameters				AST (peak) U/L	Cr (endpoint) or (peak) µmol/L	NH_3_ (endpoint) µmol/L	Complication stage (endpoint)		Survival time (h)	Endpoint
		Cell number (10^9^)	Cell viability (%)		Duration (h)				HE	AKI (or Peak)		
			48 h	54 h								
ST	1	0	0	0	0	4440	407	714	IV	III	70	Ⅳ HE
ST	2	0	0	0	0	14,750	321	431	IV	II	56	Ⅳ HE
ST	3	0	0	0	0	14,140	617	653	IV	III	54	Ⅳ HE
ST	4	0	0	0	0	3080	213	316	IV	II	72	Ⅳ HE
ST	5	0	0	0	0	9490	158	183	II	I	60	HR arrest
ST+Sham BAL	1	0	0	0	8	3520	167	581	IV	I	66	Ⅳ HE
ST+Sham BAL	2	0	0	0	4	200,000	152	362	IV	I	57	Ⅳ HE
ST+Sham BAL	3	0	0	0	8	12,300	205	265	III	II	52	HR arrest
ST+Sham BAL	4	0	0	0	8	11,440	201	344	IV	II	58	Ⅳ HE
ST+Sham BAL	5	0	0	0	8	6370	297	315	IV	II	77	Ⅳ HE
ST+BAL	1	3.6	82	73	8	4430	114 (145)	94	No	No (I)	120	Survival
ST+BAL	2	3.3	85	80	8	11,750	166	151	I	I	120	Survival
ST+BAL	3	4.2	86	72	8	11,160	179	86	No	I	80	HR arrest
ST+BAL	4	4.5	72	60	8	14,020	60 (87)	136	I	No	120	Survival
ST+BAL	5	4.6	91	77	8	12,120	71 (110)	57	No	No	120	Survival

HR arrest: nonrecoverable cardiorespiratory arrest; HE: hepatic encephalopathy.
