# Supplementary material for: Fiber scaffold bioartificial liver therapy relieves acute liver failure and extrahepatic organ injury in pigs
Source: Theranostics. 2021 Jun 11;11(16):7620–39. doi: 10.7150/thno.58515 (PMC8315066; doi:10.7150/thno.58515)
Supplement: Supplementary file 1 — Supplementary figures and tables. [file thnov11p7620s1.pdf]

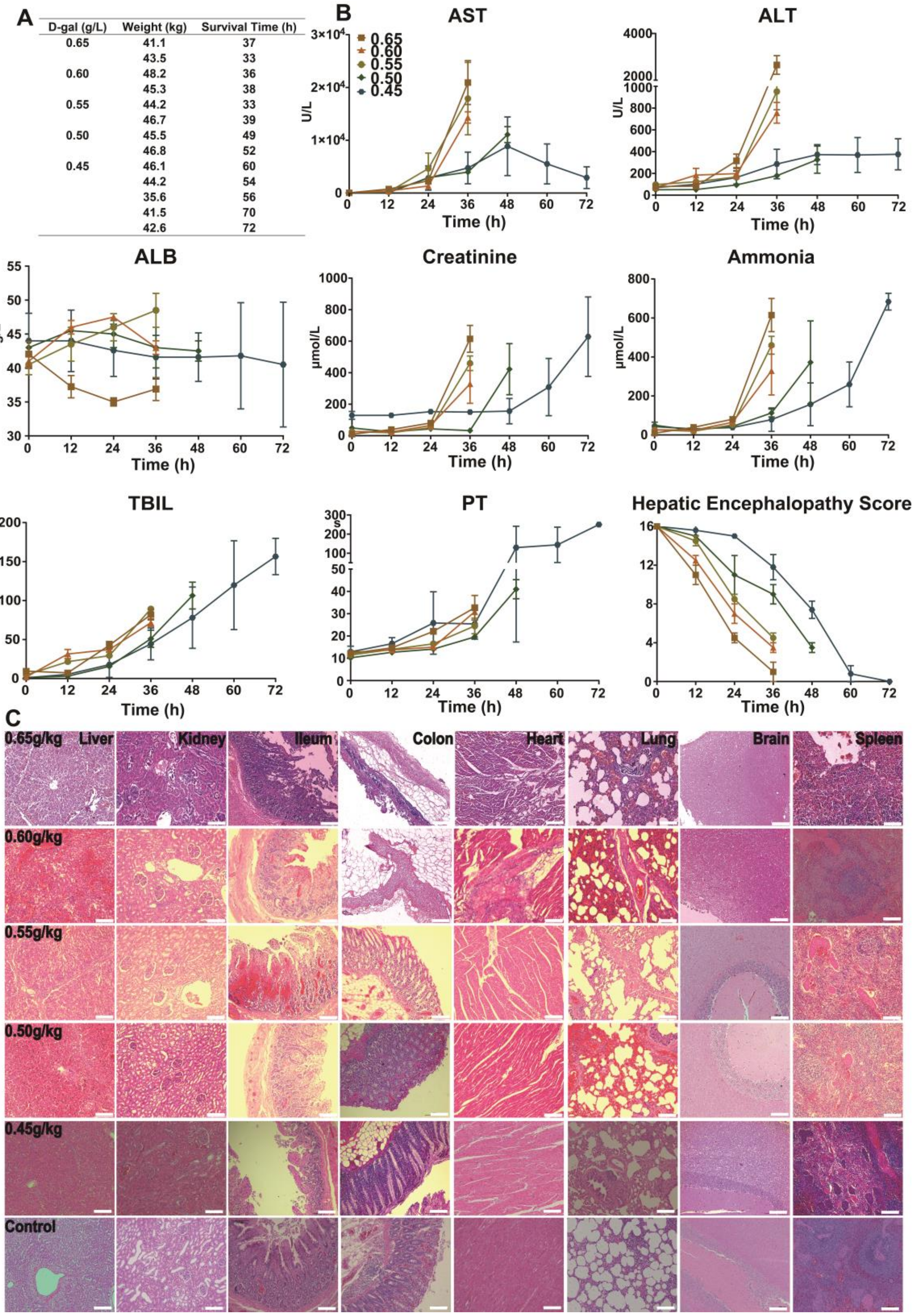

Supplemental Figure 1 Establishing a D-gal-induced Tibetan miniature pig ALF model

A. Tibetan miniature pigs (35.6-46.7 kg) were used for dose selection experiments. D-gal dose and survival time are shown.

B. The blood chemicals ALT, AST, ammonia, ALB, Cr, and TBIL and the PT and HE score were examined every 12 h.

C. Tissues, including those from the liver, kidney, ileum, colon, heart, lung, brain and spleen, were subjected to H&E pathological examination after animal sacrifice (Bar = 200 μm).

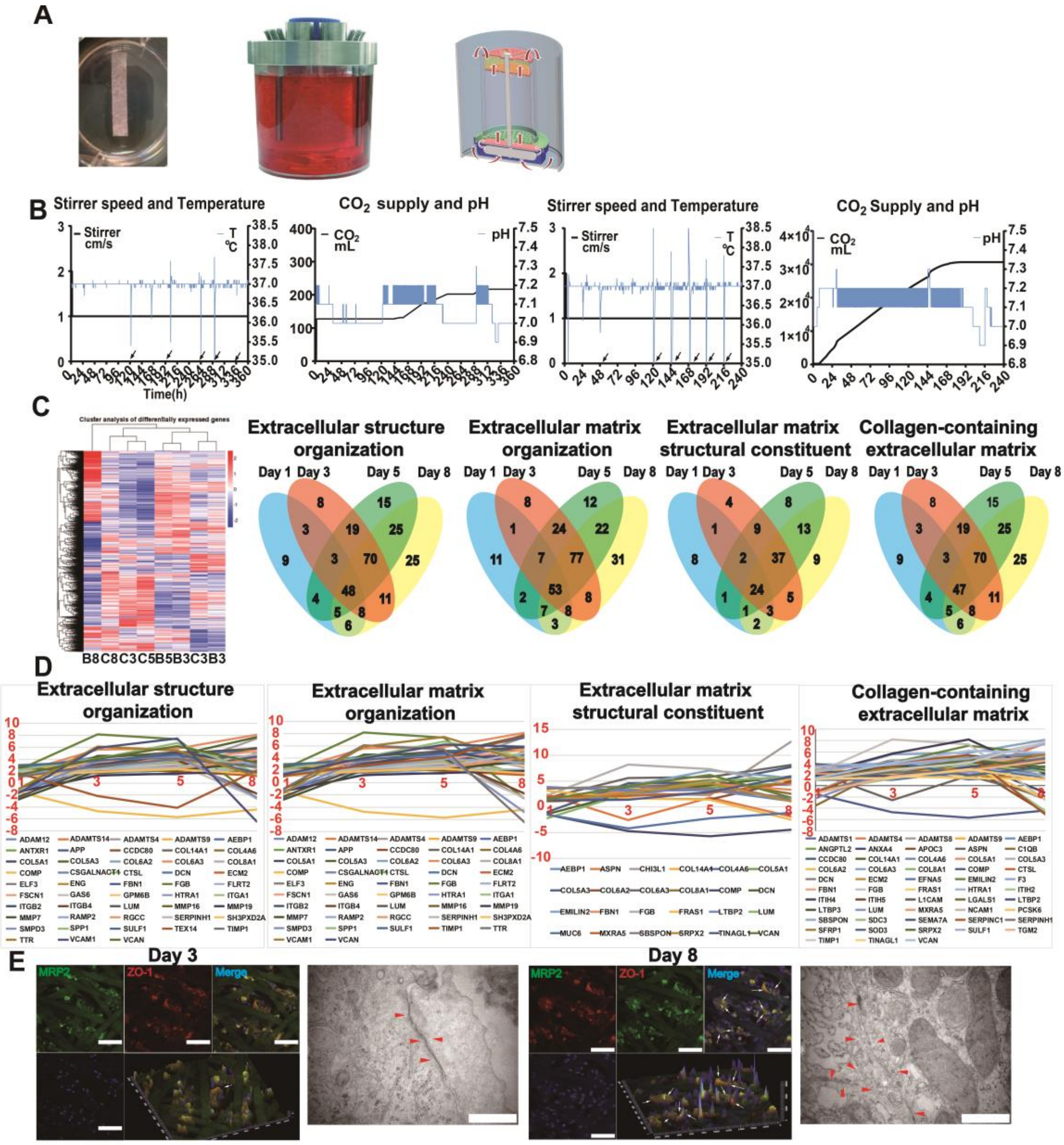

Supplemental Figure 2 Bioreactor 3D culture of C3A cells and PHHs

A. Fiber scaffold (left), bioreactor containing 4 m^2 of fiber scaffold (middle), medium circulation from bottom to top with flow down through the outer wall to exchange O_2 and CO_2 (right).

B. Stirrer speed, temperature, pH level and CO_2 supply during C3A cell (left) and PHH (right) culture (arrows indicate medium exchange).

C. RNA-seq detection of PHHs cultured in bioreactors (B) and flasks (Control, C) after 1, 3, 5 and 8 d (left). GO pathway enrichment of differentially expressed genes (DEGs) between bioreactor and flask cultures after 1, 3, 5 and 8 d. Extracellular structure organization, ECM organization, ECM structural constituents and collagen-containing ECM pathways remained activated during culture. Pathways of DEGs maintained during culture selected by Venn

diagrams.

D. Pathways of DEGs expressed during culture, except COMP, showed different levels of upregulation during culture.

E. Immunofluorescence of PHH MRP2 (bile duct protein) and ZO-1 (tight junction protein) expression. ZO-1 at both ends and MRP2 in the middle represent a bile duct-like structure (white arrow, Bar = 100 μm), which was identified by TEM (red arrow, Bar = 700 nm) on day 3. A longer and more complicated bile duct-like structure was observed on day 8.

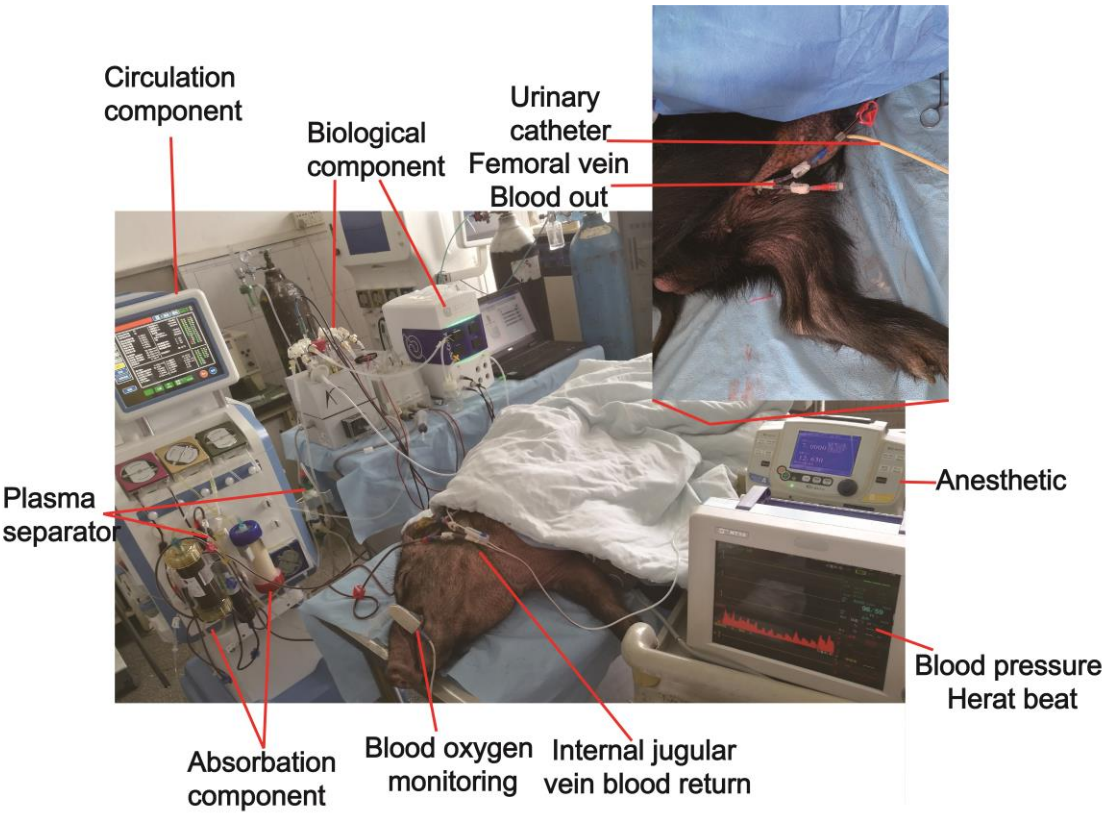

Supplemental Figure 3 BAL treatment in an ALF porcine model

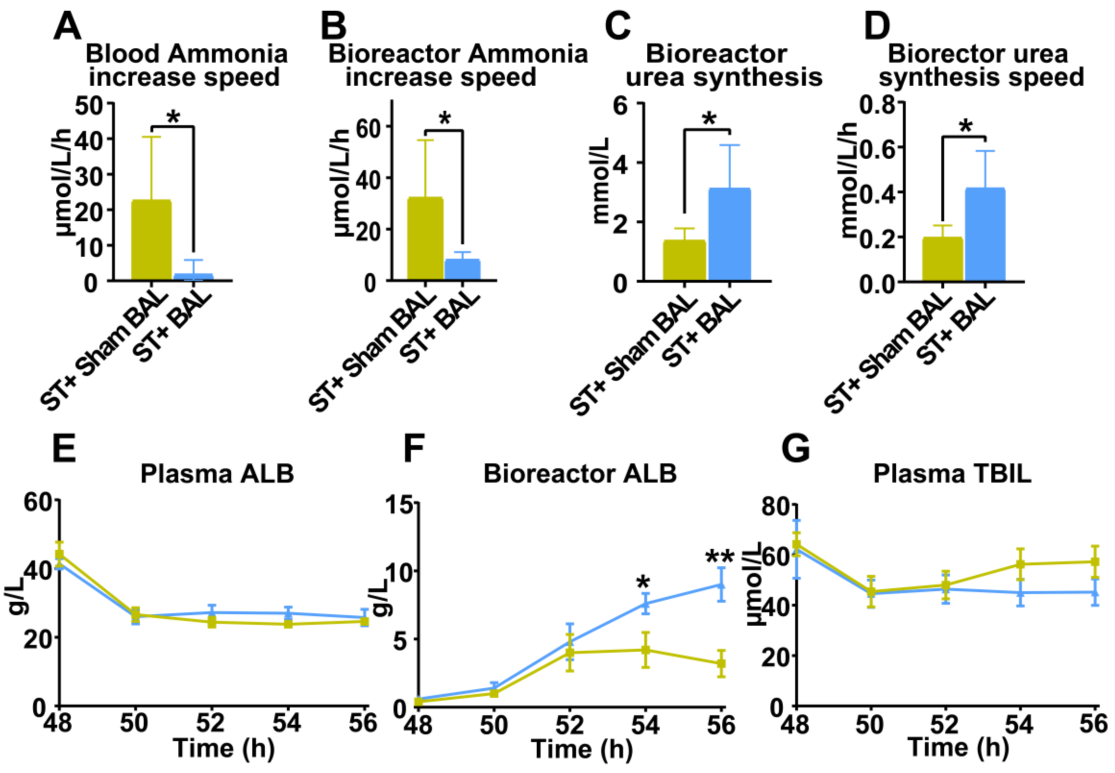

Supplemental Figure 4 BAL treatment in an ALF porcine model

A. Blood ammonia increased speed during BAL treatment (n = 5 per group, P < 0.05).

B. Bioreactor ammonia increased speed during BAL treatment (n = 5 per group, P < 0.05).

C. Bioreactor urea synthesis during BAL treatment (n=5 per group, P < 0.05).

D. Bioreactor urea synthesis speed during BAL treatment (n=5 per group, P < 0.05).

E. Plasma ALB change during BAL treatment (n = 5 per group).

F. Blood ALB change during BAL treatment (n = 5 per group, P < 0.05, P < 0.01).

G. Plasma TBIL change during BAL treatment (n = 5 per group).

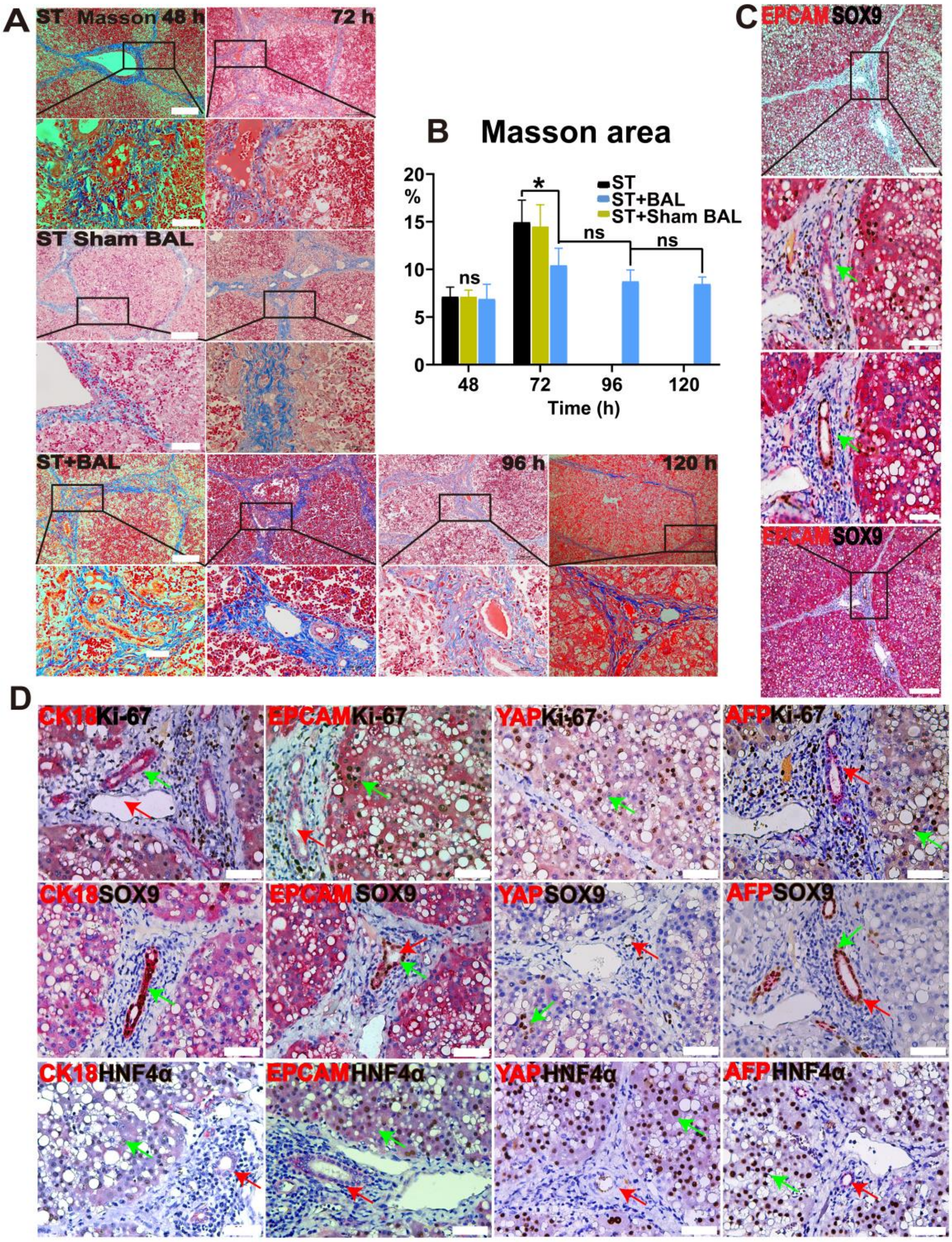

Supplemental Figure 5 Liver pathology and regeneration

A-B. Masson's staining of liver tissue from all groups (higher magnification images are shown below insets). The positive area per 40X field was calculated for at least 4 separate tissue sections per pig ($p < 0.05$, Bar = 200 μm, Magnification Bar = 50 μm).

C. Serial section of liver from the ST+BAL group after immunostaining for Ki-67, Epcam and SOX9 at 120 h (Green arrows represented serial section, Bar = 50 μm).

D. Ki-67, SOX9, HNF4α, CK18, Epcam, YAP, AFP co-immunostaining of liver from the ST+BAL group at 120 h (Green arrows represented co-immunostaining tissue, red arrows represented bile duct, Bar = 50 μm).

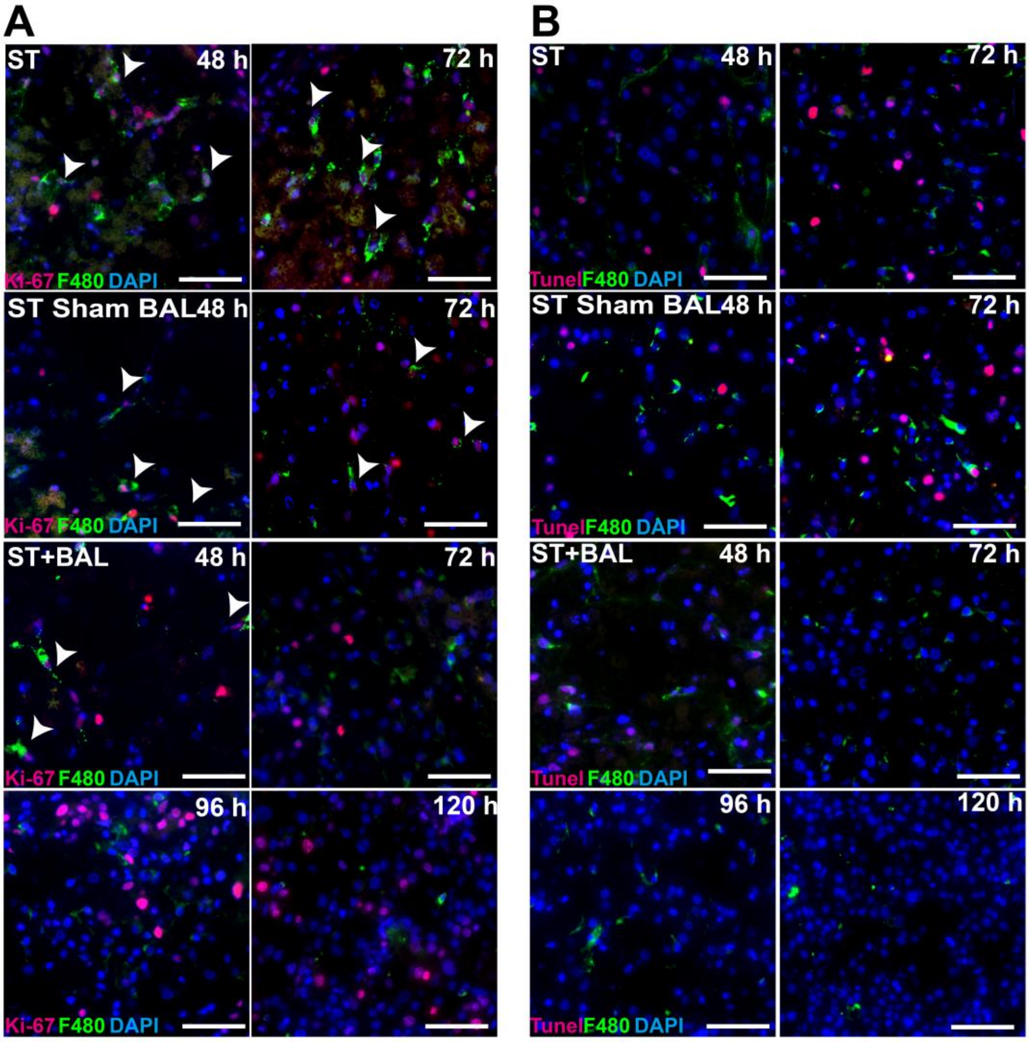

Supplemental Figure 6 Kupffer cells in BAL treatment

A. Ki67 and F480 staining of Kupffer cells in ST, ST+ShamBAL and BAL group (white arrow for o-staining, Bar = 40 μm)

B. Tunel and F480 staining of Kupffer cells in ST, ST+ShamBAL and BAL group (Bar = 40 μm)

Supplement Table 1 mRNA primers

UGT1A1	NM_000463.2	CCCACTGTATTCTTCTTGCA	GGCAATGAGCATGTTCTTC	
GCK	NM_000162.3	AGGACGTAATGCGCATCACT	TCCGACTCGATGAAGGTGATC	
G6PC	NM_000151.3	GGAATCCAGTAACTCAGTGTCA	CTGCACCCAGGATGAAATAA	
ALB	NM_000477.5	GCCCTGTGCAGAAGACTATC	GGGAACGTATGTTTCATCGA	
TF	NM_001063.3	TGAATGCAAGCCTGTGAAGT	TAGACAAACCCTCCATCCAA	
APOA1	NM_000039.1	ACCTAAAGCTCCTTGACAACTG	ACCTCCTCCAGATCCTTGCT	
AAT	NM_000295.4	CTGGGCATCACTAAGGTCTTC	ACATGGGTATGGCCTCTAAA	
ALT	NM_005309.2	CACCTACCACTTCCGGATGA	GTGAACTTGGCATGGAACCT	
GOT2	NM_001286220.1	TGGCTGCAAGAAGTGAAAGT	TTCAGGCTTTAGCCCTGTGA	
LDHA	NM_001135239.1	GGTGGTTGAGAGTGCTTATGA	GCAAGGAACACTAAGGAAGAC	
ALPL	NM_000478.4	CAAGCACTCCCACTTCATCT	TTGTTCCTGTTCAGCTCGTA	H
CPS1	NM_001122633.2	AAGGATGCTACCCGGAAGA	CAATGAAGTCAACCCCAAGA	u
GLUL	NM_001033044.2	CCAATTCCTACCTTCGTTCA	GCTACCTCGCACTGGTAAGA	m
CYP3A4	NM_001202855.2	AAAGTCGCCTCGAAGATACA	GAGAACACTGCTCGTGGTT	an
CYP2D6	NM_000106.5	ACCACTGCCGTGATTCATG	GGTTGGTGATGAGTGTCGTT	
CYP1A2	NM_000761.4	TCTTTGGAGCAGGATTTGAC	CTGGATCTTCCTCTGTATCTCA	
GSTA1	NM_145740.3	AGCTCCACTACTTCAATGCA	GCACTTGCTGGAACATCAA	
F2	NM_000506.3	TCCTGGAATCCTACATCGA	GGTCATTCTCGGTGAAGTTC	
F5	NM_000130.4	TGGAAACCATACAGGCTGAA	CCAGGCGAAGTGCAATACTT	
F7	NM_000131.4	CACCCACAGTTGAATATCCA	CCCAGATGGTGTTGATCAG	
PXR	NM_003889.3	TCCTCTTCCGAGCTGCTTTG	AGAATGATGTGGCCGGAATG	
β-actin	NM_001101.3	TGGATCAGCAAGCAGGAGTA	TCGGCCACATTGTGAACTTT	
E.coli1		GTTAATACCTTTGCTCATTGA	ACCAGGGTATCTAATCCTGTT	
E.coli2		CATTGACGTTACCCGCAGAAGAAGC	CTCTACGAGACTCAAGCTTGC	
UGT1A1	XM_003483776	GTTTATTGGTGGTGGGATCAACTGCG	CCCAAAGCATCAGCAATTTCCATAG	
GCK	XM_013985832.2	GGCTATTTCTGCCTTGGAAGC	CTGCTCCGCCTTTTCCTTCTTG	
ALB	NM_001005208.1	TCAAGAAGCAAACTGCACTCG	AGGCTAAGATCCCTCGAATTTCA	
TF	NM_001244653.1	TTGGCCAGAGTTGGCAAAAC	GGCTTCCAGGAGTTTTGAGGT	
APOA1	NM_214398.1	ACCTCAACCTGAAACTCCTGG	GTCCTTGCTCATCTCCTGCC	
ALT	XM_005662806.3	CACAGCTGCCACTTCAGTCC	GGCCATGACCCTTGCACCT	
GOT2	NM_213928.1	CCCTAGTGTCCGAAAGGCAG	CCCGGTCCCAGAAATGGTC	P
LDHA	NM_001172363.2	GGGGGAGTACCGGAGCA	GGATTTAGAACCAAAAGGAATCGGG	i
CPS1	XM_005672159.3	AAAGAACAGTGGTGAGCTGAGT	GATTTGTGAAAAGAGTAAGCTGGT	g
GLUL	NM_213909.1	GGTCTGAAGTACATCGAGGAGGC	AATCCAGTTAGGCGCCGTGT	
CYP2D25	NM_214394.1	CGCTCCGAAGGAGTGATCC	CCCACTCCTCCAACGACTTC	
CYP1A2	NM_001159614.1	CACAGCGAGGAGAATTCCAGCA	GTGGTAATTGTATCAAATCCGGCTC	
GSTA1	NM_214389.2	GCTGAAGGCCCTGAAAACCAG	TGTCCTGGGACACTCAGCC	
F2	NM_001122985.1	ACCTGAAGGAGACCTGGACA	TCAGGCTTGTAACCAGCACA	
F5	NM_214120.1	GACGGCAGGGAGGAGCA	GCAAAAGGGCTTGAACTGGG	
F7	NM_001044591.1	TGGGTTTCCTACAACGACGG	GTTGGTTTCGCAGTTCCGAC	
PXR	NM_001038005.1	ACCACCAAGCAGTCCAAGAG	ATAACCAGTGGCCTTGTCCC	

GAPDH	NM_001206359.1	TCGGAGTGAACGGATTTGGC	TGACAAGCTTCCCGTTCTCC	

Supplement Table 2 Circulation device component information

Component	Brand	Model number
Peristaltic pump		
P1	PreFluid	TH162
P2	PreFluid	TH152
P3	PreFluid	TH153
P4	PreFluid	TH154
Bubble detection sensor	Solenoids China	AD101
PH sensor	AppliSens	Z010011025
PO_2 sensor	AppliSens	Z001012051
Circulation pipe	Baihemedical	8mm*12mm
Computer control and rest parts are designed and manufactured by our group		

Supplement Table 3 Medium circulation speed, volume and magnetic impeller speed

Circulation speed (cm/s)	Total volume (ml)	Magnetic impeller (RPM)
1	1000	673
1	900	741
1	800	809
2	1000	1043
2	900	1069
2	800	1094

Supplement Table 4 Waterfall Height and Medium working volume

Waterfall Height (cm)	Dip tube length (cm)	Medium working Volume (mL)
1	9	1004
2	8	937
3	7	870
4	6	802
5	5	675
6	4	568
